# Global chromosome rearrangement induced by CRISPR-Cas9 reshapes the genome and transcriptome of human cells

**DOI:** 10.1093/nar/gkac153

**Published:** 2022-03-04

**Authors:** Ying Liu, Guangwei Ma, Zenghong Gao, Jian Li, Jin Wang, Xiangping Zhu, Ruowu Ma, Jiawen Yang, Yiting Zhou, Kaishun Hu, Yin Zhang, Yabin Guo

**Affiliations:** Guangdong Provincial Key Laboratory of Malignant Tumor Epigenetics and Gene Regulation, Guangdong-Hong Kong Joint Laboratory for RNA Medicine, Medical Research Center, Sun Yat-sen Memorial Hospital, Sun Yat-sen University, Guangzhou 510120, China; Guangdong Provincial Key Laboratory of Malignant Tumor Epigenetics and Gene Regulation, Guangdong-Hong Kong Joint Laboratory for RNA Medicine, Medical Research Center, Sun Yat-sen Memorial Hospital, Sun Yat-sen University, Guangzhou 510120, China; Ministry of Education Key Laboratory for Ecology of Tropical Islands, Key Laboratory of Tropical Animal and Plant Ecology of Hainan Province, College of Life Sciences, Hainan Normal University, Haikou 571158, China; Guangdong Provincial Key Laboratory of Malignant Tumor Epigenetics and Gene Regulation, Guangdong-Hong Kong Joint Laboratory for RNA Medicine, Medical Research Center, Sun Yat-sen Memorial Hospital, Sun Yat-sen University, Guangzhou 510120, China; Guangdong Provincial Key Laboratory of Malignant Tumor Epigenetics and Gene Regulation, Guangdong-Hong Kong Joint Laboratory for RNA Medicine, Medical Research Center, Sun Yat-sen Memorial Hospital, Sun Yat-sen University, Guangzhou 510120, China; Guangdong Provincial Key Laboratory of Malignant Tumor Epigenetics and Gene Regulation, Guangdong-Hong Kong Joint Laboratory for RNA Medicine, Medical Research Center, Sun Yat-sen Memorial Hospital, Sun Yat-sen University, Guangzhou 510120, China; Guangdong Provincial Key Laboratory of Malignant Tumor Epigenetics and Gene Regulation, Guangdong-Hong Kong Joint Laboratory for RNA Medicine, Medical Research Center, Sun Yat-sen Memorial Hospital, Sun Yat-sen University, Guangzhou 510120, China; Guangdong Provincial Key Laboratory of Malignant Tumor Epigenetics and Gene Regulation, Guangdong-Hong Kong Joint Laboratory for RNA Medicine, Medical Research Center, Sun Yat-sen Memorial Hospital, Sun Yat-sen University, Guangzhou 510120, China; Guangdong Provincial Key Laboratory of Malignant Tumor Epigenetics and Gene Regulation, Guangdong-Hong Kong Joint Laboratory for RNA Medicine, Medical Research Center, Sun Yat-sen Memorial Hospital, Sun Yat-sen University, Guangzhou 510120, China; State Key Laboratory of Biocontrol, School of Life Sciences, Sun Yat-sen University, Guangzhou 510275, China; Guangdong Provincial Key Laboratory of Malignant Tumor Epigenetics and Gene Regulation, Guangdong-Hong Kong Joint Laboratory for RNA Medicine, Medical Research Center, Sun Yat-sen Memorial Hospital, Sun Yat-sen University, Guangzhou 510120, China; Guangdong Provincial Key Laboratory of Malignant Tumor Epigenetics and Gene Regulation, Guangdong-Hong Kong Joint Laboratory for RNA Medicine, Medical Research Center, Sun Yat-sen Memorial Hospital, Sun Yat-sen University, Guangzhou 510120, China; Guangdong Provincial Key Laboratory of Malignant Tumor Epigenetics and Gene Regulation, Guangdong-Hong Kong Joint Laboratory for RNA Medicine, Medical Research Center, Sun Yat-sen Memorial Hospital, Sun Yat-sen University, Guangzhou 510120, China; Guangdong Provincial Key Laboratory of Malignant Tumor Epigenetics and Gene Regulation, Guangdong-Hong Kong Joint Laboratory for RNA Medicine, Medical Research Center, Sun Yat-sen Memorial Hospital, Sun Yat-sen University, Guangzhou 510120, China

## Abstract

Chromosome rearrangement plays important roles in development, carcinogenesis and evolution. However, its mechanism and subsequent effects are not fully understood. Large-scale chromosome rearrangement has been performed in the simple eukaryote, wine yeast, but the relative research in mammalian cells remains at the level of individual chromosome rearrangement due to technical limitations. In this study, we used CRISPR-Cas9 to target the highly repetitive human endogenous retrotransposons, LINE-1 and Alu, resulting in a large number of DNA double-strand breaks in the chromosomes. While this operation killed the majority of the cells, we eventually obtained live cell groups. Karyotype analysis and genome re-sequencing proved that we have achieved global chromosome rearrangement (GCR) in human cells. The copy number variations of the GCR genomes showed typical patterns observed in tumor genomes. The ATAC-seq and RNA-seq further revealed that the epigenetic and transcriptomic landscapes were deeply reshaped by GCR. Gene expressions related to p53 pathway, DNA repair, cell cycle and apoptosis were greatly altered to facilitate the cell survival. Our study provided a new application of CRISPR-Cas9 and a practical approach for GCR in complex mammalian genomes.

## INTRODUCTION

**Figure 1.**
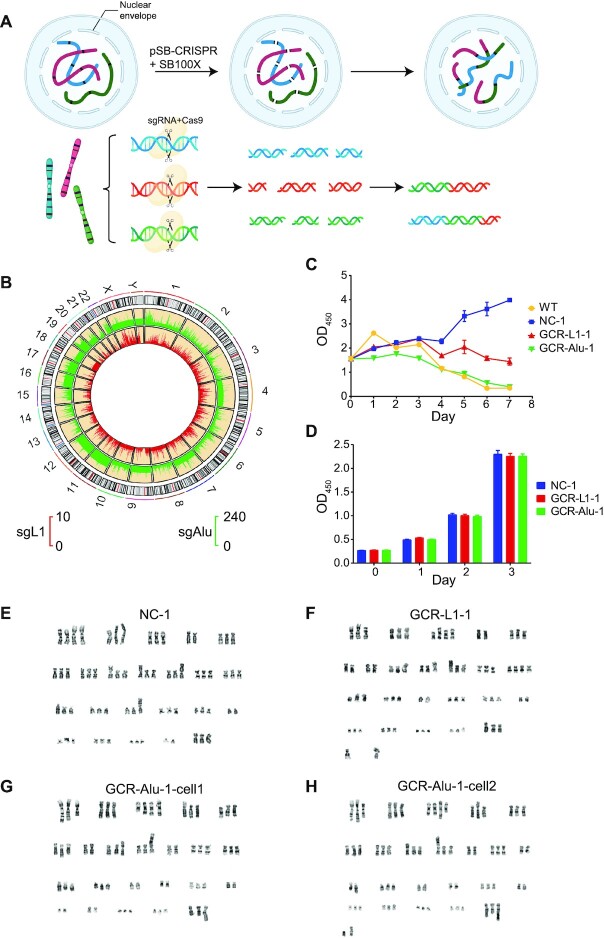
The design of CReaC, cell selections and cytogenetic assay. (**A**) The mechanism that CReaC works. (**B**) The distributions of sgL1 and sgAlu on the human chromosomes, the red bars of the inner track is for sgL1 and green bars of the outer track is for sgAlu. (**C**) The growth rate that the cells were transfected at day0 and selected in the presence of puromycin at day1. WT (wild type 293T) is the pool of cells that wasn’t transfected by plasmid with puromycin resistance. (**D**) The growth rate of the cells after selection for one month. (E–H) karyotype of NC-1 (**E**), GCR-L1-1 (**F**) and GCR-Alu-1 (**G**, **H**).

Chromosome rearrangements are mutations that cause genomic structural variations, including insertions, deletions, duplications, copy-number variations (CNVs), inversions and translocations. Chromosome rearrangements are usually caused by DNA double strand breaks (DSBs) and rejoins (1). It is known that 0.5% of neonatal genomes have abnormalities caused by chromosome rearrangements (2). The famous Robertsonian translocations occur between the human acrocentric chromosomes (chr13, 14, 15, 21, 22 and Y), which may have normal phenotype, but sometime can cause Downs syndrome or other diseases (3). Chromosome rearrangements also contribute for carcinogenesis (4). For example, the Philadelphia chromosome is a rearrangement between chromosome 9 and 22, which makes ABL1 and the strong promoter of BCR (break point cluster region) fuse to form BCR-ABL chimeric gene, resulting in continuous high activity of ABL1 kinase and cell transformation (5). Moreover, Chromosome rearrangement is a major motivation for evolution (6). Chromosome rearrangements not only change the primary structure of DNA, but also change the three-dimensional (3D) conformation of chromatins (7). For example, Shao et al. combined 16 chromosomes of *Saccharomyces cerevisiae* into one, creating a yeast strain with only one chromosome in 2018 (8), and later they further cyclized this large chromosome into a circular chromosome, like a typical prokaryotic chromosome (9). This process greatly changed the 3D structure of the yeast genome. But surprisingly, the growth of this strain (with singular linear chromosome) is similar to that of the wild type (8). Of course, its gene expression profile has changed significantly. Also in yeast, Jef Boeke team developed a method called SCRaMbLE, in which loxP sites are inserted into synthetic chromosomes and Cre recombinase is used to trigger chromosome rearrangements (10–12). Many interesting results have been obtained using this method, which provides a great facility for the study of chromosome structure (including 3D structure) and function (13). As multicellular organisms face many more challenges, such as cell differentiation, development and homeostasis, the mammalian genomes are more sophisticatedly regulated at both 3D structure and epigenetics level. Obviously, if similar studies can be performed in the mammalian cells, it would be more helpful for us to understand the roles of chromosome structure in the development and diseases. However, mammalian genomes are far larger and more complex than the yeast genome and a method to induce massive chromosome rearrangements is yet absent, which limits the research in this field. In recent years, CRISPR-Cas9 genome editing technology has made great progress. Under the guidance of sgRNA, Cas9 endonuclease specifically cleaves DNA sequences, and the cleaved DNA strands were subsequently rejoined via non-homologous end joining (NHEJ) pathway (14,15). There are large numbers of repetitive sequences in the mammalian genomes (16). If sgRNAs are designed according these sequences, a large number of chromosome breaks can be generated, which will lead to global chromosome rearrangements (GCR) (Figure 1A). Here, we developed a method called Chromosome Rearrangement by CRISPR-Cas9 (CReaC). We used sgRNAs to target endogenous retrotransposons, LINE-1 (L1) or Alu in HEK293T cells and obtained cells with significant different karyotypes from the control cells. Whole genome sequencing (WGS) showed that large numbers of inversions, translocations and CNVs have occurred. Then we further performed transcriptomic and epigenetic studies to evaluate the effect of the GCR on the cell physiological status.

## MATERIALS AND METHODS

### Cells culture

The human immortalized normal renal cell line HEK 293T was obtained from the American Type Culture Collection (ATCC) and maintained in Dulbecco's modified Eagle's medium (DMEM, Gibco) supplemented with 10% fetal bovine serum (Gibco) and penicillin-streptomycin (HyClone) in a humidified incubator containing 5% CO_2_ at 37°C.

### Plasmid construction, transfection and cell selection

The sgRNAs, sgL1 (TTCCAATCAATAGAAAAAGA) and sgAlu (TGTAATCCCAGCACTTTGGG) were designed according to the sequence of the conserved regions of L1 and Alu respectively. The exact match number of these sgRNAs were searched against hg38 genome using a Perl script and confirmed using bowtie2 with the parameter of ‘–no-1mm-upfront -a’. The sites with one or two mismatch were searched using bowtie with the parameter of ‘-a -v 1’ or ‘-a -v 2’ (17) (Supplementary Table S1; Figure S1A). The sgRNA sequences were cloned into pSB-CRISPR vector (18), and a sgRNA sequence with no match in hg38 (CGCTTCCGCGGCCCGTTCAA) from the GeCKO library Human_GeCKOv2_Library_B_1 of the previous study (15) was used as negative control, the sgNC. Next, pSB-CRISPR and SB100X plasmids at a ratio of 10:1 were transfected into 293T cells using Lipofectamine 3000 (Invitrogen) following the manufacturer's protocol. After 24 h the transfected cells were selected with puromycin at 1 μg/ml (Solarbio) for up to four weeks. The cells that survived were designated as GCR-L1, GCR-Alu and NC, respectively.

RCC2 knockout in GCR-L1, GCR-Alu and NC cells were also established using pSB-CRISPR system with sgRNA sequences, sgRCC2#1, TTGTGTCTGCAGCATGTGGGCGG and sgRCC2#2, TGCAGTAGCAGCAGCGGCGG, as previously described (18), and selected with Blasticidin S (Solarbio) at 10 μg/ml concentration for 3 weeks.

### Cell proliferation assay

Cell proliferation was measured using Cell Counting Kit-8 (CCK-8) assays. Briefly, 3 × 10^3^ GCR-L1, GCR-Alu and NC cells suspensions were seeded in a 96-well plates and were cultured for 24 h, 48 h and 72 h. A total of 10 μl of CCK-8 solution (APExBIO) was added to each well for 2 h-incubation at 37°C at the same time every day, and then the absorbance at 450 nm was recorded using an enzyme immunoassay analyzer (TECAN Spark 10M).

### Survival rate after transfection

1 × 10^4^ 293T cells suspensions were seeded in a 96-well plates for 24 h, and then pSB-CRISPR-sgNC, pSB-CRISPR-sgL1 and pSB-CRISPR-sgAlu plasmids were transfected together with SB100X plasmid into the cells using Lipofectamine 3000 (Invitrogen) following the manufacturer's protocol. After 24 h the transfected cells were selected with puromycin at 1 μg/ml (Solarbio). CCK-8 assays as above were performed to detect cells survival rates at the 0, 24, 48, 72, 96, 120, 144, 168 h after selection.

### RNA synthesis detection

Cell-Light assay, based on combination of EU and Apollo fluorescent dyes, was used to detect cells RNA synthesis. 1 × 10^5^ GCR-L1, GCR-Alu and NC cell suspensions were seeded in 12-well plates for 24 h. Cells were fixed and stained for new synthesized RNA following the protocol of the Cell-Light EU Apollo567 RNA Imaging Kit (Ribobio), and then stained for DNA using DAPI. Finally, the images were observed and recorded using a fluorescence microscope (Olympus IX71, Tokyo, Japan).

### Cytogenetics analysis

Cytogenetics analysis of GCR-L1, GCR-Alu, NC were performed using G-banding techniques. Briefly, the cells were incubated with 0.06 μg/μl of colcemid for 2.5 h at 37°C, and then trypsinized, resuspended, centrifuged. The cells were then incubated in 0.075 M potassium chloride for 30 min at 37°C and fixed with Carnoy's solution 3:1 (acetic acid:methanol). The metaphase chromosomes were analyzed for G-banding (500 band level) by Guangzhou LanGuang Co., Ltd. At least five cells were analyzed for each group of cells, and abnormality recognition and karyotype nomenclature were performed as the International System for Human Cytogenetic Nomenclature (ISCN).

### Quantitative reverse transcription PCR (qRT-PCR)

The total RNA samples of GCR-L1, GCR-Alu, NC cells were collected using Trizol Reagent (Invitrogen, #15596026), and then reverse-transcribed using the PrimeScript RT Reagent Kit (Takara RR047A). The real-time PCR was performed using SYBR Premix ExTaq™ (Takara) analyzed by Light Cycler® 96 real-time PCR thermocycler (Roche). The qPCR primers are listed as following: Puro-F, GATCCGGACCGCCACATC; Puro-R, CCACACCTTGCCGATGTC, Cas9-F, CAGATTCGCCTGGATGACCA; Cas9-R, ATCCGCTCGATGAAGCTCTG; GAPDH-F, CCATGGGGAAGGTGAAGGTC; GAPDH-R, GAAGGGGTCATTGATGGCAAC. The expression was first normalized to GAPDH transcripts and then the comparative Ct (ΔΔCT) analysis method was used to calculate the fold change between the NC and GCR groups.

### Western blot

Western blot were performed as previously described (19). Briefly, the cells were collected and lysed with RIPA buffer (50 mM Tris–HCl [pH 8.0], 5 mM EDTA, 150 mM NaCl and 0.5% Nonidet P-40) and a protease & phosphatase cocktail (Bimake, Shanghai, China). Equal amounts of protein were separated by 10% SDS-PAGE gel and transferred onto a polyvinylidene difluoride (PVDF) membranes (Millipore). The membranes were incubated with RCC2 (CST, #5104), Flag (Sigma-Aldrich, #F1804) and GAPDH (CST, #2118) antibodies overnight at 4°C. Secondary antibodies (Transgen Biotech, #HS101) were incubated for 1 h at room temperature. The membranes were visualized using ECL detection reagents (Beyotime #P0018A, Shanghai, China).

### Whole genome resequencing and somatic variation analysis

Next generation sequencing (NGS) library preparations were constructed following the manufacturer's protocol (NEBNext^®^ Ultra™ DNA Library Prep Kit for Illumina^®^). The libraries were sequenced using the Illumina HiSeq instrument with PE150 configuration by Genewiz Co., Ltd, Guangzhou, China. The clean data were aligned with human genome hg38 using BWA (version 0.7.12) (20). CREST (21) and Control-FREEC (version 10.6) (22) were used to analyze the genomic structure variations.

### Transcriptome sequencing and analysis

Total RNA of the GCR-L1, GCR-Alu and NC cells was isolated using Trizol reagent (Invitrogen) according to the manufacturer's instructions. RNA integrity and quantity were finally measured using RNA Nano 6000 Assay Kit (Agilent) of the Bioanalyzer 2100 system. Ribosomal RNA was removed from total RNA, followed by fragmenting RNA into short fragments of 250–300 bp, and strand-specific libraries were constructed using the dUTP method (23). Through screening fragment length (about 200bp), all RNAs except ribosomal RNA and small fragment RNA (microRNA, siRNA, etc.) were finally obtained, including lncRNA, mRNA, circular RNA (circRNA). The libraries were prepared and sequenced using Illumina sequencing by Novogene Co., Ltd, Beijing, China. The raw reads were processed by removing the adaptor reads and low-quality tags. Clean reads for each sample were mapped to hg38 using the software HISAT2 (24). FPKM, the number of fragments per kilobase of gene sequence per millions base pairs sequenced was used to quantify the expression levels of a mRNA or lncRNA. The differential expression analysis of two conditions was performed using the edgeR R package (version 3.22.5). The *P* values were adjusted using the Benjamini & Hochberg method. Corrected *P* value <0.05 and absolute foldchange >2 were set as the threshold for significantly differential expression.

The circRNAs were identified by integrated analysis using find_circ (25) and CIRI (26), the read counts of junction sites were then analyzed by DEGseq. The expression level was normalized with TPM (transcript per million).

### ATAC-seq library preparation and sequencing

ATAC-seq seq (Assay for Transposase-Accessible Chromatin with high-throughput sequencing) was performed, according to the published protocol (27). Briefly, when GCR-L1, GCR-Alu and NC cells were grown to 70–80% confluence, 5 × 10^5^ viable cells were lysed in 10 mM Tris–HCl (pH 7.4), 10 mM NaCl, 3 mM MgCl_2_, and 0.1% (v/v) Igepal CA-630 and the nucleus was extracted. Transposition reaction was performed using TruePrep® DNA Library Prep Kit (Vazyme) at 37°C for 30 min, followed by purifying immediately. The libraries were amplified for 15 cycles using TruePrep^®^ DNA Library Prep Kit (Vazyme), and sequenced using Illumina NovaSeqTM 6000 by Guangzhou Gene Denovo Biotechnology Co., Ltd., Guangzhou, China. After removing adapters and low quality reads, Bowtie2 (17) with the parameters –X2000 and –m1 was used to align the clean reads from each sample against hg38 genome assembly, and the reads aligned to the mitochondrial genome were filtered. Peaks were called using MACS2 (version 2.1.2) (28) with parameters ‘–nomodel –shift -100 –extsize 200 -B -q 0.05’. The DiffBind was used to analyse peak differences across groups, significant differential peaks were filter with FDR <0.05 in two comparison groups. Peak related genes and the distribution of peak on different genome regions (such as promoter, 5′UTR, 3′UTR, exon, intron, downstream and intergenic) were determined using ChIPseeker (version v1.16.1) (29).

### ONT library preparation and sequencing

We used the PromethION platform with Ligation Sequencing Kit (SQK-LSK109) from ONT Company to construct 1D library. After passing the quality inspection, the DNAs were fragmented and then the enrichment and purification of the long DNA fragment (the initial filter length was 15 kb) were performed. The purified DNA were subjected to end repair, purified again and ligated with ONT-standard sequencing adapters, motor proteins and Tether proteins. The prepared DNA library was sequenced with the PromethION platform. The official ONT software Guppy (https://timkahlke.github.io/LongRead_tutorials/BS_G.html) was used to perform basecalling. Then the raw data was stored in the FASTQ file format. Subsequently, Nanopack software package (30) was used to filter pass reads with better quality than Q7, and to remove reads with length shorter than 500bp. The final data was called clean reads. The clean data was aligned to the reference genome (GRCh38/hg38) using minimap2 (31).

### The identification of the translocations from the ONT sequencing reads

The clean sequencing reads were aligned to the L1 or Alu sequences (from https://dbrip.brocku.ca/dbRIPdownload/) using minimap2, and sequencing reads containing L1/Alu elements were extracted from the output files. Then, these sequences were mapped to the hg38 human genome and the structures with L1/Alu elements flanked by sequences from different chromosomal parts were considered as translocations. The scripts for extracting sequences and filtering the alignments were written in Perl language.

### The analysis on copy number variations

**Figure 2.**
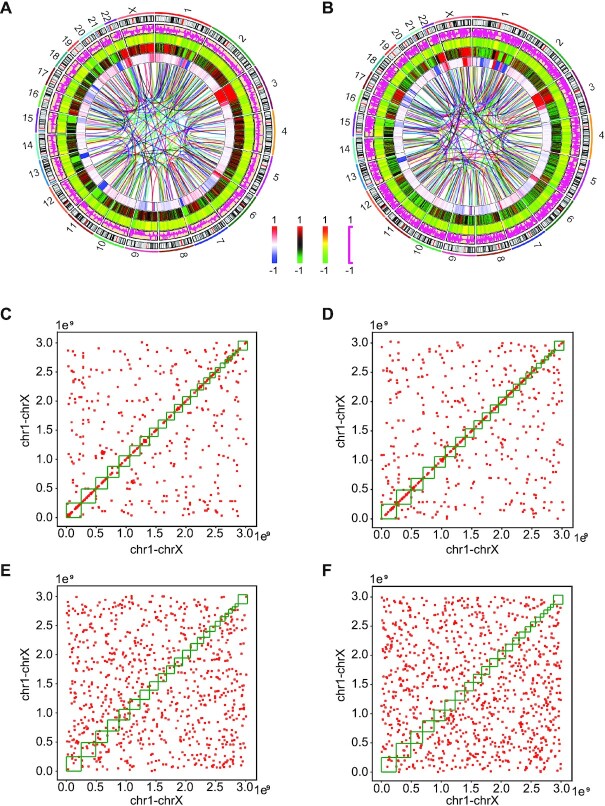
Multi-omics analyses were performed to the GCR and NC cells. (A, B) The chromosomal structure, chromatin accessibility and gene expression changes of GCR-L1-1 versus NC-1 (**A**) and GCR-Alu-1 versus NC-1 (**B**). The tracks of the Circos charts from the center to the outer are translocation/inversion, difference of CNV, difference of raw sequence match of ATAC-seq, difference of ATAC-seq peaks and the difference of gene expression. The interval for the heatmaps and curves is 500 kb. (C, D) 2D plots show the intra- and inter-chromosome translocations in GCR-L1-1 (**C**) and GCR-Alu-1 (**D**). The x- and y-axes represent the linearly arranged haploid genome (chr1, chr2…chr22, chrX). The x and y values represent the genomic loci of the two breakpoints of the translocation. (**E**, **F**), the 2D plots of Monte Carlo (MC) simulations for GCR-L1-1 (C) and GCR-Alu-1 (D).

**Figure 3.**
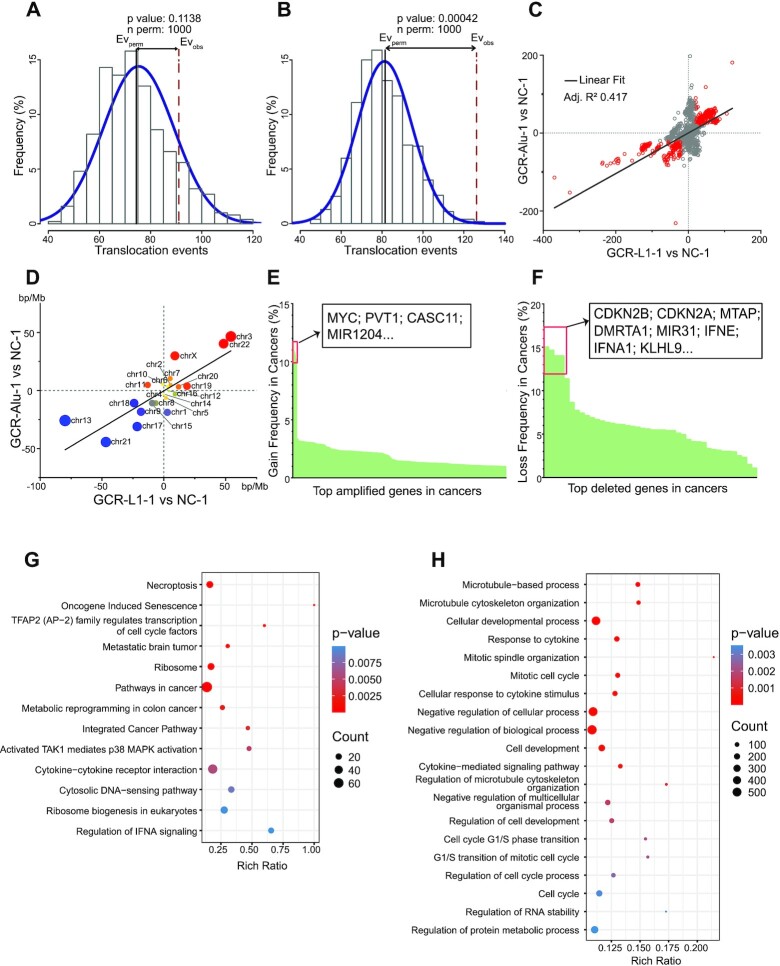
The copy number variations of the GCR cells. (A, B) The translocation events in the high CNV regions of GCR-L1-1 versus NC-1 (**A**) and GCR-Alu-1 versus NC-1 (**B**) the genomic regions of 500 kb (same size for calculating CNV) were permutated 1000 times to assess the distribution of translation events of random region. Normal distribution was used to calculate the *P* value of observed translations in the high CNV regions. (**C**) The correlation the CNVs/500 kb-interval between GCR-L1-1 versus NC-1 and GCR-Alu-1 versus NC-1, the red points indicate sequence read matches significantly changed with same trend in the two GCR cells. (**D**) The correlation of genomic gain and loss at the chromosome-level between GCR-L1-1 versus NC-1 and GCR-Alu-1 versus NC-1. (E, F) the gain (**E**) and loss (**F**) frequency in clinical tumor samples of the genes at the high CNV regions shared by GCR-Alu-1 versus NC-1 and GCR-L1-1 versus NC-1, which was calculated using TCGA clinical samples data obtained from Cosmic. (G, H) The high CNVs shared by GCR-L1-1 versus NC-1 and GCR-Alu-1 versus NC-1 were analyzed using WikiPathway, Reactome, KEGG (**G**) and GO (**H**) databases.

The genome was divided into equal intervals of 500 kb, and the differences of sequence read matches of WGS were calculated. The differences of matches per interval between GCR-L1 or GCR-Alu and NC represent the CNVs (Figures 2A and B; 3C). The regions with high CNVs in both GCR-L1 and GCR-Alu are the common high CNV regions. To analyze the translocation events in the common high CNV regions, each translation site were intersected with CNV coordinates using bedtools, and the translocation/inversion sites located in the common high CNV regions were counted. For random regions, the regions with the same bin size of each CNV regions were shuffled 1000 times using bedtools, the amounts of translocation sites that these random regions were from each time were counted, and the distribution of translocation events were assessed (Figure 3A and B). The genes in the common high CNV regions were annotated using Refseq annotation. Similarly, the TCGA clinical CNV data from Cosmic were also annotated using Refseq. The frequency of each genes observed in cosmic data were then calculated (Figure 3E and F).

### Monte Carlo Simulation for the translocations (for Figure 2C–F; Supplementary Figure S3)

To see if intra-chromosomal translocations or inter-chromosomal translocations were preferred during the GCR, Monte Carlo (MC) simulations were performed. Random chromosomal positions with the same numbers as the experimental data were generated using a Perl script. The script was run for ten times to avoid bias and the average numbers of intra- or inter-chromosomal translocations were used to compare with the experimental data. The *P* values were determined by chi-square test. The points on the charts of the MC simulation (Figure 2E and F; Supplementary Figure S3C) were from one of the ten runs of the Perl script.

### Calculation of the distances from translocation/inversion points to the nearest L1/Alu elements

The loci of L1 and Alu in hg38 were from the RepeatMasker annotation (https://genome.ucsc.edu/cgi-bin/hgTables). For each of the translocation breakpoints, the loci of the relative elements (L1 or Alu) on the same chromosome were scanned, the closest elements were determined, and the distance from the breakpoints to the closest elements were calculated. The script was written in Perl language.

### Functional enrichment analysis

The Gene Ontology (GO), KEGG pathway, WikiPathway, Reactome and TRRUST enrichment analysis of differentially expressed genes (DEGs) were implemented by the clusterProfiler R package, CPDB (cpdb.molgen.mpg.de/CPDB), and metascape (https://metascape.org). Pathway terms with corrected *P* value <0.05 were considered significantly enriched. The enrichment results were visualized by Cytoscape, plug-in Bingo and ggplot package in RStudio (32). The enriched terms with a similarity >0.3 were connected by edges and we selected the terms with the best *P*-values from each of the 20 clusters where term labels were only shown for one term per cluster, shown in Figure 5F and Supplementary Figure S9B. GSEA was performed to identify the Hallmark pathways (33,34). Protein-protein interaction (PPI) analysis was carried out with STRING, BioGrid and the Molecular Complex Detection (MCODE) algorithm (35) was applied to identify densely connected network components. Pathway and process enrichment analysis was applied to each MCODE component independently, and the best-scoring terms by *P*-value have been retained as the functional description of the corresponding components, shown in Supplementary Figure S9C.

### Other bioinformatics analyses and charts

The heatmaps of hierarchical cluster (H-cluster) were generated using pheatmap R packages with hierarchical clustering method. The scatter plots (Figure 2E and F; Supplementary Figure S2) were generated using matplotlib package in Python. The Circos charts (Figure 2A and B) were generated using RCircos package in RStudio. Briefly, the genome was divided into equal intervals of 500 kb, and the differences of sequence read matches of WGS, RNA-seq and ATAC-seq, and the peak coverages of ATAC-seq between GCR-L1 or GCR-Alu and NC were plotted as heatmaps or curves. The links at the center of charts were plotted according to the somatic structure variation data. Scripts for bioinformatics analyses were written in Perl, Python or R languages. The pictures of Figures 1A and 8 were created with the aid of BioRender (biorender.com).

## RESULT

### Experiment design and cell selection

There are >500 000 LINE-1 (intact elements and fragments) and >1 000 000 Alu copies in the human genome (16), which provides an ideal set of targets for making multiple DNA DSBs using CRISPR-Cas9. We designed several sgRNAs according to the conserved regions of L1 and Alu (Supplementary Table S1), and two of them, sgL1 and sgAlu, were chosen for the present study, which have 7398 and 317 924 matching sites respectively in the hg38 human genome haploid version (Figure 1B, Supplementary Figure S1A). sgAlu is also the sgRNA candidate with the most matching sites in the current version of human genome. Additionally, sgNC, a 20 nt sequences with no match in the human genome from the previous study (15) was used as negative control sgRNA. The sgRNA sequences were cloned into pSB-CRISPR plasmid as previously described (18). The CRISPR cassette (sgRNA, Cas9 and puromycin resistance gene) in pSB-CRISPR is flanked by the terminal repeats of the Sleeping Beauty (SB) transposon, IRDR-L and IRDR-R, and can be cleaved and integrated into the host genome by the SB transposase. The hyperactive SB100X transposase expression plasmid (36) and the pSB plasmids (pSB-CRISPR-sgNC, pSB-CRISPR-sgL1 and pSB-CRISPR-sgAlu) were co-transfected into HEK293T cells respectively. The cells were then kept in the presence of puromycin, forcing the Cas9 endonuclease and sgRNAs to express constantly and keep cleaving the chromosomes. The cells that survived after puromycin selection were designated as GCR-L1-1, GCR-Alu-1 and NC-1 respectively.

As expected, the survival rates of GCR-L1-1 and GCR-Alu-1 are lower than that of NC, due to the strong stress caused by multiple DSBs (Figure 1C). The GCR-L1-1 and GCR-Alu-1 cells recovered, and eventually grew up after roughly three weeks. Then, they started to proliferate stably, and there was no difference between the growth rates of the GCR and NC cells. Thus, new immortalized strains were created by targeting repetitive retroelements with CRISPR-Cas9 (Figure 1D).

To see if chromosome rearrangements really happened, cytogenetics assay was performed. GCR and NC cells were treated with the classical Giemsa Staining (Figure 1E–H). It is known that HEK293 cells are female human cells with a karyotype near triploid (37). Typical NC cells have 67 chromosomes as expected, while GCR cells showed apparent chromosome aberrations, and unrecognizable chromosomes were observed in both of them (Figure 1F and H). Moreover, one of the GCR-Alu-1 cells contains only 63 chromosomes (Figure 1G), indicating that CReaC might be used as a tool for genome minimization. The cytogenetics assays were carried out twice, and different karyotypes were obtained each time.

In order to understand the GCR cells more comprehensively, the CReaC operation was repeated twice and six single clones (NC-2M, GCR-L1-2M, GCR-Alu-2M, NC-3M, GCR-L1-3M and GCR-Alu-3M) were isolated from the survived GCR and NC cell pools as described in Supplementary Figure S1B. The growth rates of the single clones were also tested using CCK-8 and showed certain diversity (Supplementary Figure S1C).

### GCR-L1 and GCR-Alu cells were evaluated using multi-omics approaches

To evaluate the new selected strains comprehensively, we performed WGS, RNA-seq and ATAC-seq for the GCR and NC cells (Figure 2A and B). Since 293T cells are immortalized cells and already carry substantial burden of chromosome arrangements, all the changes in the GCR genomes were compared to those in the NC genomes instead of the reference human genome. Additionally, WGS and RNA-seq were also performed for the cells from the six single clones.

The WGS shows hundreds of translocation/inversions and apparent CNVs across the GCR-L1-1 and GCR-Alu-1 genomes (Figure 2A and B; Supplementary Table S2). The single clones of the GCR cells also showed various translocation and CNV patterns (Supplementary Figure S2A–D).

We counted the numbers of inter-chromosome translocations and intra-chromosome translocations (including inversions) respectively, and Monte Carlo (MC) simulations were also performed according to the experimental data (see Methods). The comparison showed significant different between the experimental data and the MC simulation (Supplementary Figure S3A). The intra-chromosome translocations in the GCR genomes were far more frequent than the inter-chromosome translocation. We plotted all the translocation events as well as the MC data on two-dimension charts (Figure 2C–F; Supplementary Figure S3B and C). Lots of points clustered around the diagonals, indicating that the intra-chromosome translocations manly occurred between the adjacent or nearby retroelements. The Hi-C studies showed that there are significantly more intra-chromosome interactions than inter-chromosome interactions (38). Whether the translocations are associated with the interactions between different chromosomal regions can be explored in the future study.

RNA-seq showed that the gene expression profiles of both the GCR cells were significantly different from that of NC. And it seemed that the change of the expression profile of GCR-Alu-1 was even more extensive than that of GCR-L1-1 (Figure 2A and B, pink curves).

The ATAC-seq showed that although the raw sequence reads that matched to the chromosomes were similar between the three groups (Figure 2A and B, green-black-red heatmap), the peak densities of both GCR strains were dramatically decreased compared to that of NC (green-yellow-red heatmap).

An overview of multi-omics showed that the GCR cells differ from the NC cells in multiple dimensions, including the genomic primary structure, the epigenetic modification and the gene expression profile. Detailed analyses were carried out in the following sections.

### The genomic structure variations of GCR cells

Since the GCR were induced by cleaving the repetitive elements of L1/Alu, we wondered how close the translocation breakpoints were to these repetitive elements. We calculated the distributions of the breakpoint relative to the closest L1/Alu elements (Supplementary Figure S4A). About half of the breakpoints were within L1/Alu elements or <1 kb to the closest elements. The breakpoints that were far from L1/Alu elements could have been produced by off-target effect or secondary DSBs due to the genome instability.

It is challenging for the alignment of short sequencing reads of NGS at repetitive regions. To further validate the translocations around repetitive regions, we sequenced the GCR genomes using the Oxford Nanopore's Technology (ONT, one of the third generation sequencing techniques). A number of sequencing reads with L1/Alu elements flanked by sequences from different chromosomal parts were identified and two examples were shown in Supplementary Figure S4B and C.

In the Circos chart of GCR-Alu-1 (Figure 2B), it seems that regions that contain more translocation/inversion events also contain more CNVs. To test this assumption, we compared the numbers of translocation/inversion events in the regions containing high-level CNVs to those in the randomized genomic regions (the shuffled regions of same length as those for evaluating CNVs, see Materials and Methods). As shown in Figure 3A and B, for GCR-Alu-1, the mean translocation/inversion events in randomized genomic regions is 81, while there are significantly more translocation/inversion events in the regions with high-level CNVs (126 events, *P* = 0.00042). However, similar difference was not found between the regions with high-level CNVs and the random regions in GCR-L1 (*P* = 0.1138), which might be because L1 is much longer than Alu and the subsequent recombination process are more complex.

It seems that the CNV distributions are quite similar between GCR-L1-1 and GCR-Alu-1 from the Circos chart (Figure 2A and B), so we tested the correlation between these two distributions based on the same 500 kb intervals as the Circos chart. Figure 3C showed that the *R*^2^ between the two distributions was 0.417, indicating a strong correlation. Moreover, if viewed from the chromosome level, the correlation was even stronger (*R*^2^ = 0.683, Figure 2D), which indicated that the CNVs played important roles for the survival of the cells under strong stress. The slopes of the two trend lines also showed that the CNVs in GCR-L1 are more intense than those in GCR-Alu (Figure 3C and D).

Notably, the common CNVs shared by GCR-Alu and GCR-L1 resembled many features of copy number changes that observed in clinical tumor samples, including the most frequent and important regions of gain and loss (Figure 3E and F; Supplementary Table S3). For example, the chr9p21 is the most frequent deleted region in cancer genomes, which contains the important tumor suppressors such as CDKN2A (p16), CDKN2B (p15). p15 and p16 are cyclin-dependent kinase (CDK) inhibitors, that maintain the active state of Rb family members, and promote their binding to E2F1 (39), leading to G1 cell cycle arrest (40). The loss of p15 and p16 helps the tumor cells bypassing the G1 cell cycle arrest. Moreover, previous study revealed that p16 is required for the reduction in CDK4- and CDK6-mediated Rb kinase activity upon DNA damage (41). Since the NHEJ repair mainly happens during G1 phase (42), the simultaneous deletion of Chr9p21 in both GCR cell groups suggests that the loss of p16 and p15 may be a key step for the cells to survive the G1 arrest induced by the multiple NHEJ repairs. Besides, we found the most common amplification of Chr8q24 in tumors also occurred in GCR-L1 and GCR-Alu genomes. This region contains the most famous oncogene, MYC, as well as the onco-lncRNA, PVT1. It is reported that deregulated c-Myc disables the p53-mediated DNA damage response and helps cells with damaged genomes to bypass cell arrest and enter the cell cycle (43).

The genes in the high-level CNV regions were analyzed using KEGG, Reactome, WikiPathway and Gene Ontology (GO) enrichments. Figure 3G and H showed that typical pathways related to cell survival such as the ‘cell cycle’, ‘G1/S transition’ and ‘pathways in cancer’ were enriched, which also indicated the contributions of the CNVs to the survival of the GCR cells.

Similar analyses were also performed for the WGS of the single clones (Supplementary Figure S5). Some well known oncogenes, such as AKT3, and tumor suppressors, such as RB1 and PARK2 emerged in the high or low CNV regions. In brief, while the single clones showed certain diversity, they also share a series of common oncogene amplifications and tumor suppressor deletions.

### Large-scale chromosome rearrangements reshaped the landscape of gene expression

**Figure 4.**
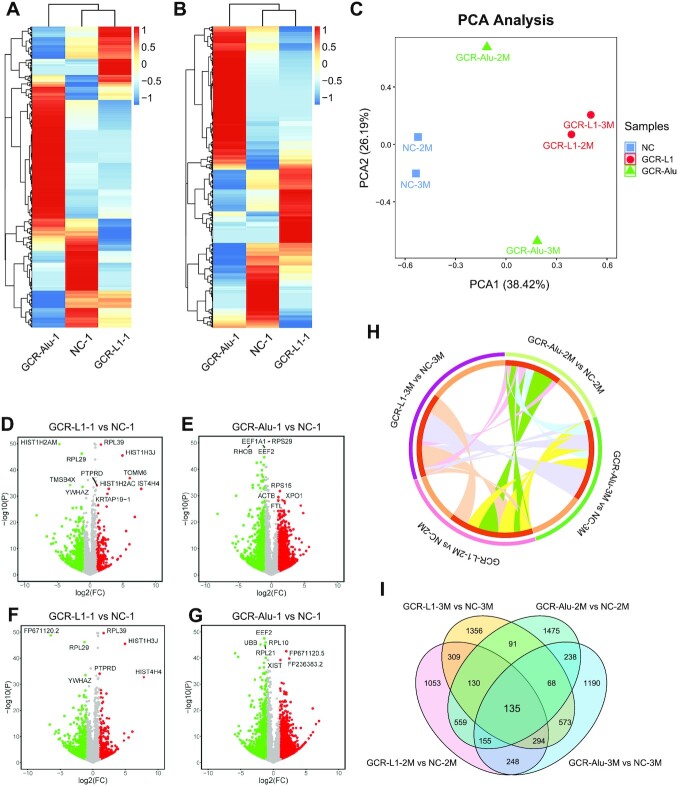
The global transcriptome profiling of GCR and NC cells. (A, B), H-cluster of expression at gene-level of mRNAs (**A**) and lncRNAs (**B**). (**C**) PCA analysis of transcriptome of six single clones. (D) Volcano plots show the DEGs of mRNA (**D**. **E**) and lncRNA (**F**, **G**) in GCR-L1-1 versus NC-1 (**C**, **E**) and GCR-Alu-1 versus NC-1 (**D, F**). (**H**) The overlaps between top 3000 DEGs from four set of DEGs are shown as a Circos plot. I, Venn diagram of four sets of DEGs.

The gene expressions of the GCR cells were compared with the NC cells at mRNA level (Figure 4A, D–E) and lncRNA level (Figure 4B, F–G; Supplementary Table S4). Similar to the sequencing read coverage showed in Figure 2A and B, both of the gene expressions of GCR-L1 and GCR-Alu were changed greatly. There were 425 mRNAs upregulated and 965 downregulated in GCR-L1-1, and 1947 mRNAs upregulated and 1,920 downregulated in GCR-Alu-1 (Figure 4D and E). Similarly, considerable numbers of lncRNAs changed as well (Figure 4F and G). Genes of ribosome RNAs, ribosome proteins and histones were found in DEGs with the lowest *P* values, indicating the chromatin structure and the peptide translation were greatly altered in the GCR cells. The change of gene expression is greater in GCR-Alu than that in GCR-L1, which is also consistent to the RNA-seq read coverage (Figure 2A and B; Supplementary Figure S6I).

When it came to monoclonal cell lines, GCR cells revealed more DEGs than cell pools did (Supplementary Figure S6H). Principal Component Analysis (PCA) shows that NC cell replicates were very tight as well as replicates of GCR-L1 were at mRNA expression level, while the replicates of GCR-Alu present very different from each other (Figure 4C). H-cluster of mRNA expression level and Pearson correlation coefficients come to the similar conclusion (Supplementary Figure S6G, J). These results confirmed that GCR induced by CReaC possesses randomness. However, there are a lot of overlaps among four sets of DEGs when GCR cells were compared to NC cells as shown in Figure 4H, and 135 genes were shared among four comparisons (Figure 4I), which indicates that transcriptome changes induced by CReaC share similarities despite the randomness, and probably accounts for cell survival under stress.

Compared to the expression changes at gene-level, the expression changes at transcript-level are even more drastic (Supplementary Figure S6A–F). The number of differential alternative splicing (AS) was shown in Supplementary Figure S7A. The differential AS number of GCR-Alu-1 versus NC-1 is significantly greater than that of GCR-L1-1 versus NC-1 (*P* = 0.038, Supplementary Table S5). It is reported that both L1 and Alu contribute for AS and it seems that the function of Alu on AS is even stronger that that of L1 (44–47). AS of RNA plays important roles in the gene expression regulation of eukaryotes (48) and aberrant AS is an important contribution for carcinogenesis (49). Therefore, AS might be a major contribution for the GCR cells to survive the severe stress.

Besides the expressions of mRNAs and lncRNAs, we also examined the expression of circRNAs of cell pools. There is a significant decrease of circRNA abundance in GCR-Alu-1 of which the abundance is only ∼2/3 of that in NC-1 (Supplementary Figure S7B; Table S6). It is known that Alu elements are important for the formation of circRNAs. The homologous sequences of Alu at the flanking introns help the RNA molecules cyclize into circRNAs (50,51). Therefore, the damage at Alu elements may result in reduced circRNA formation.

The circRNA expressions between cell groups also vary greatly (Supplementary Figure S7C–E). The changes in GCR-L1 and GCR-Alu showed a fairly large overlap (Supplementary Figure S7F). The KEGG enrichment of the host gene of differentially expressed circRNAs in the two groups also shared multiple pathways. Protein processing in endoplasmic reticulum, ubiquitin mediated proteolysis, RNA transport and cell cycle are the most significant enriched pathways, which also points to the degradation of the abnormal proteins and the response to stress (Supplementary Figures S7G and S7H).

### Multiple pathways associated to cell survival were altered in GCR cells

**Figure 5.**
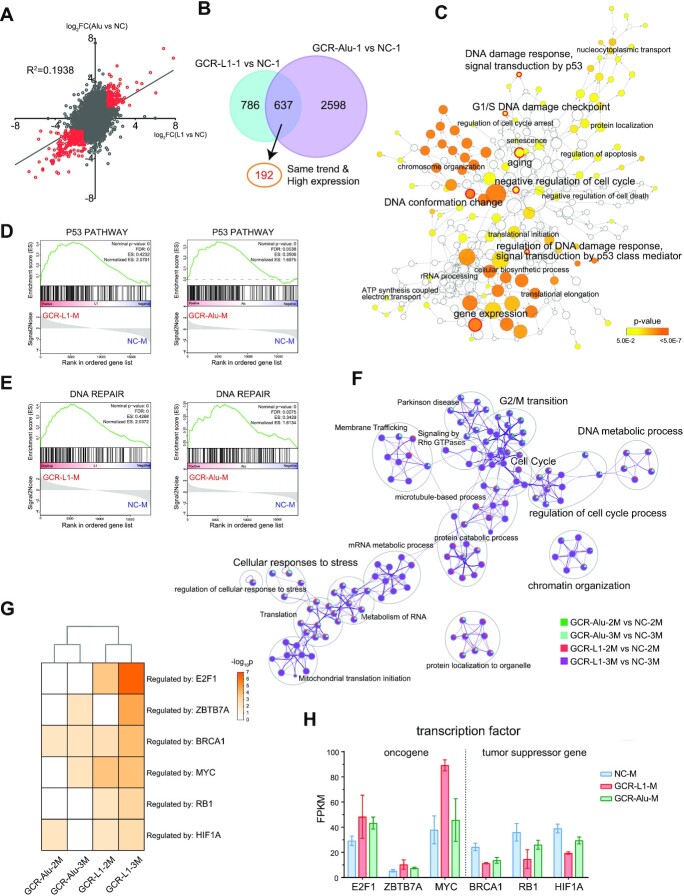
Multiple pathways associated with cell survival were altered in GCR cells. (**A**) The correlation of expression fold changes at gene-level between GCR-L1-1 versus NC-1 and GCR-Alu-1 versus NC-1 (*R*^2^= 0.1938). The red points indicate the genes whose expression changed with same trend in the two GCR cells and with fold change ≥2. (**B**) The numbers of the genes that marked red in panel A. (**C**) GO enrichment analysis of genes marked red in 5A at gene-level shown as an interaction network using Cytoscape plug-in, BinGO. The hierarchical relationships of terms are connected by arrows. Terms with *P*< 0.05 are colored. (**D**), GSEA Enrichment plot: p53 pathway profile of the running ES Score and positions of geneset members on the Rank Ordered List. (**E**) GSEA Enrichment plot: DNA repair profile of the running ES Score and positions of geneset members on the Rank Ordered List. (**F**) Functional enrichment analysis result are visualized as networks plot where terms are collected and grouped into clusters based on their membership similarities. The terms with the best *P*-values from each of the clusters are shown. The nodes are represented as pie charts, where the pie charts are color-coded based on the groups identities, where the size of a slice represents the percentage of genes under the term that originated from the corresponding groups. (**G**) Transcription factors enrichment analysis using TRRUST database. (**H**) Expression level of genes shown in (G).

Although GCR-Alu showed very different gene expression profile from GCR-L1, the two GCR strains still shared a lot of common changes, which might be account for the cell survival through severe stress. Figure 5A showed a weak correlation of expression changes at gene-level between GCR-L1-1 versus NC-1 and GCR-Alu-1 versus NC-1 in a scatter plot (*R*^2^ = 0.1938). The points in the first and third quadrants are the genes that changed with same trend in two GCR cells. Then the genes with |log_2_FC| > 1 (the red points in Figure 5A) and FPKM > 1 were extracted, resulting in a set of 192 genes (Figure 5B, Venn diagram).

Since the expression changes at transcript-level are more drastic than those at gene-level in GCR cells, we also examined the common transcript changes in GCR-L1-1 and GCR-Alu-1. The correlation of expression changes at transcript-level between two GCR cells is slightly stronger than those at gene-level (*R*^2^ = 0.2059, Supplementary Figure S8A). We focus on those genes whose transcript-level fold change has at least 2-fold increase or 0.5-fold decrease versus corresponding gene-level fold change (red points in Supplementary Figure S8B and C). The transcript changes of these genes showed a fairly strong correlation with an *R*^2^ of 0.5779 between two GCR cells (Supplementary Figure S8D), indicating that the changes of AS were more consistent than those of the overall gene expressions. Therefore, changes in AS may contribute more to cell survival under stress than overall changes in gene expression. Similar to the analysis in Figure 5B, we still chose the genes with |log_2_FC| > 1 at transcript-level and with FPKM >1, and a set of 520 genes (Supplementary Figure 8E) were obtained.

We then performed the functional enrichment analysis using GO (Figure 5C, Supplementary Figure S8F), as well as Reactome and WikiPathway (Supplementary Figure S8G, H) for the genes selected in Figure 5B and Supplementary Figure S8E. Notably, almost all the fundamental cellular processes, the DNA synthesis (replication), the RNA synthesis (transcription) and the protein synthesis (translation initiation and extension), were altered in GCR cells. The most significant enriched pathways were p53 and DNA damage repair, cell cycle and mitotic check points, apoptosis and some cancer related pathways, which are closely related to the cell survival under stress. The ubiquitination pathway is the major protein post translation modification, which should be due to the large quantity of defect proteins brought by the chromosome rearrangements. This result indicates that GCR has a great physiological impact on cells, so that the cells have to modify almost all the fundamental pathways to survive the crisis.

As expected, DEGs from GCR single clones were enriched in similar biological process and pathways as GCR cell pools. For example, p53 pathway, DNA repair and apoptosis that appeared in Figure 5C are also significantly up-regulated in GCR single clones via GSEA (Figure 5D, E, Supplementary Figure S10A–C). Gene-sets with stronger enrichment at GCR-L1 compared to GCR-Alu are present in most of the functional groups (Figure 5F, Supplementary Figure S9A). Although four GCR cell lines present different pattern in clusters, all of them are enriched in terms related to cell cycle. The overlapped 135 genes among four sets of DEGs (Figure 4I) also show GCR cells with damaged genomes have consistent transcriptome alterations in bypass cell cycle arrest (Figure 5F, Supplementary Figure S9B). The seed gene CCND1 in MCODE networks of DEGs has been shown to interact with tumor suppressor protein Rb, and seems to be a pan-cancer actor (Supplementary Figure S9C) (52).

The p53 pathway is particularly important for the cells upon DNA damage, and it is reported that cells with TP53 mutations are more likely to survive during CRISPR-Cas9 editing (53), so we investigated the expression of the different AS isoforms of TP53 in the GCR cells. Supplementary Figure S10D showed that the important isoforms, TP53-201, 209, 215, 219, 225 and 227, were all downregulated in GCR cells, whereas, TP53-204, 228 were upregulated. The proteins of latter two isoforms are N-terminus truncated versions, which can compete with the functional p53 and inhibit apoptosis (54–57). This result indicated that the AS of TP53 might have played a key role for the survival of GCR cells.

Furthermore, we performed TRRUST analysis and identified six most significant key regulators for the DEGs from the single clones (Figure 5G). We looked at the expressions of those transcription factors (TF), and found that oncogenes EZ2F1, ZBTB7A and MYC were all up-regulated, and tumor suppressor genes BRCA1, RB1 and HIF1A were all down-regulated in various degrees (Figure 5H), suggesting that GCR cells with reshaped transcriptome present cancerogenesis-like changes.

### GCR reshaped the chromatin accessibility

**Figure 6.**
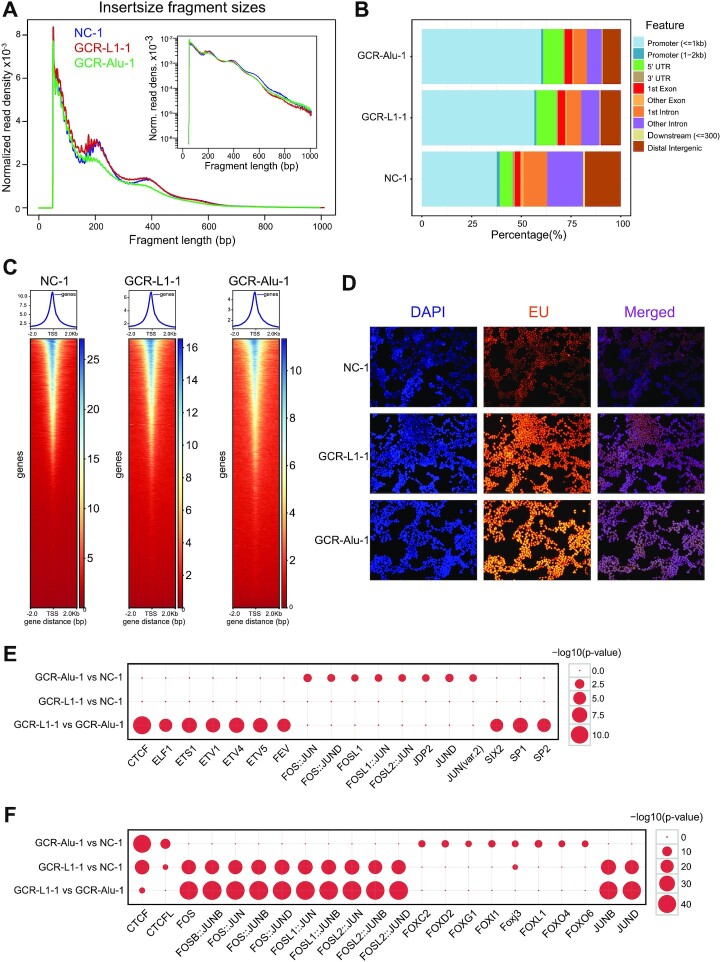
ATAC-seq revealed the change of chromatin accessibility of the GCR cells. (**A**) The insert size distributions of the NC and GCR cells. (**B**) The peak distributions in different genomic regions. (**C**) The accessibilities flanking gene TSSs. (**D**) EU staining to detect the new RNA synthesis. (E, F) The bubble diagrams of significantly up-regulated motifs (**E**) and down-regulated motifs (**F**), the abscissa is the TF name, and the ordinate is the comparison between two samples. The size of the circle indicates the –log_10_ (*P*-value) of the corresponding TF motif.

On the one hand, epigenetics plays an important role in the regulation of gene expression, while on the other hand, we also wondered about the effect of changes in the primary structure of chromosomes on epigenetic modification. Therefore, we performed ATAC-seq for the GCR and NC cells. The overview of the peak distribution changes were shown in Figure 2A and B. Here, we further discuss the experimental results in more detail. The insert fragment size analysis indicates the distribution of nucleosomes on chromatins. NC-1 showed a typical wave with a period of 200 bp, while the wave amplitude of GCR-L1-1 moderately decreased, and even more significantly, the wave of GCR-Alu-1 was almost flat (Figure 6A). This suggests that the nucleosome distribution in the GCR cells becomes less regular compared to the control cells. Presumably, the alteration in the nucleosome distribution would have broad impacts on the chromatin accessibility and the gene expression.

The ATAC-seq data was also processed using PCA (Supplementary Figure S11A). The correlation analysis showed that the chromatin accessibility of GCR-Alu-1 was more different from NC-1 than GCR-L1-1 was (Supplementary Figure S11B; Supplementary Table S7), which was consistent with the conclusion of transcriptome sequencing in Figure 4A-B and Supplementary Figure S6A, B and I. The proportion of chromatin open regions in different positions, such as promoter, exon, intron, 5′UTR, 3′UTR, etc. also changed significantly. Only <40% of the open regions in NC are located in the promoter, while more than half of the open regions in GCR-L1 and GCR-Alu are located in the promoter regions (Figure 6B). Moreover, statistics of the peak distribution flanking the gene transcription start sites (TSS) showed that nearly 80% of peaks distributed in the 1 kb window flanking the TSS in the GCR cells, but only ∼50% peaks in the same window in NC cells (Supplementary Figure S11C). Although the overall open regions in the GCR cells decreased, they became more clustered in the promoter regions. These results suggested that the gene expression of cells after chromosome recombination might have become more active compared to control cells.

We then viewed the openness at the TSS. Nearly 170 000 genes (including mRNA and lncRNA) were stacked together, and the heat map was drawn with ATAC peak values within 2 kb upstream and downstream of the TSS (Figure 6C). The three heatmaps look similar, but the scales are very different. The data ranges of the GCR cells are narrower than that of the NC cells. The diversity of the TSS openness is much larger in NC cells, whereas, the difference of openness between the high-open TSS and low-open TSS became smaller, and the open range near TSS of the high open genes become relatively wider in GCR cells. This result also indicated the disorder of the nucleosome distribution and gene-expression regulation.

Since ATAC-seq only measures the relative accessibility of chromatin and indicates the change of transcriptional activity, but not the actual RNA synthesis intensity, we performed the new RNA synthesis intensity assay in the three groups of cells. EU (5-ethynyl-2′uracil nucleoside) is a nucleoside analogue, which can be inserted into the newly formed RNA and emit fluorescence through conjugation reaction with fluorescent dye. Thus, the newly formed RNA can be detected by fluorescence microscopy or flow cytometry. Figure 6D showed that the fluorescence increases from NC-1 to GCR-L1-1 and then to GCR-Alu-1, which was what we had expected, because chromosome rearrangement inevitably led to a lot of wrong RNA products, and cells still need enough ‘right’ RNA to ensure normal physiological function, which is bound to increase the total RNA. Therefore, the cells after GCR were in the state of active RNA synthesis and degradation.

To further analyze which transcription factors were related to the different peak distributions, motif analysis was conducted for the increased and decreased peaks respectively (Figure 6E and F). Interestingly, CTCF was the top transcription factor related to the peak decrease in both GCR cells. CTCF is an insulator protein and is critical for maintaining the topologically associating domain (TAD) structure (58). In the GCR cells, the chromatin accessibility of the CTCF binding sites was decreased, indicating that the binding between CTCF and chromosomal DNAs was blocked and the formation of TAD was impaired, which is consistent to recently reported that L1 and Alu repeats blueprint the chromatin macrostructure and TAD (59). For the regions with increased peaks in GCR-Alu-1, JunD was the most remarkable transcription factor, which is also consistence to previously reported that the upstream regions of Alu tend to be associated with JunD (60).

### Cas9 was silenced in the GCR cells

**Figure 7.**
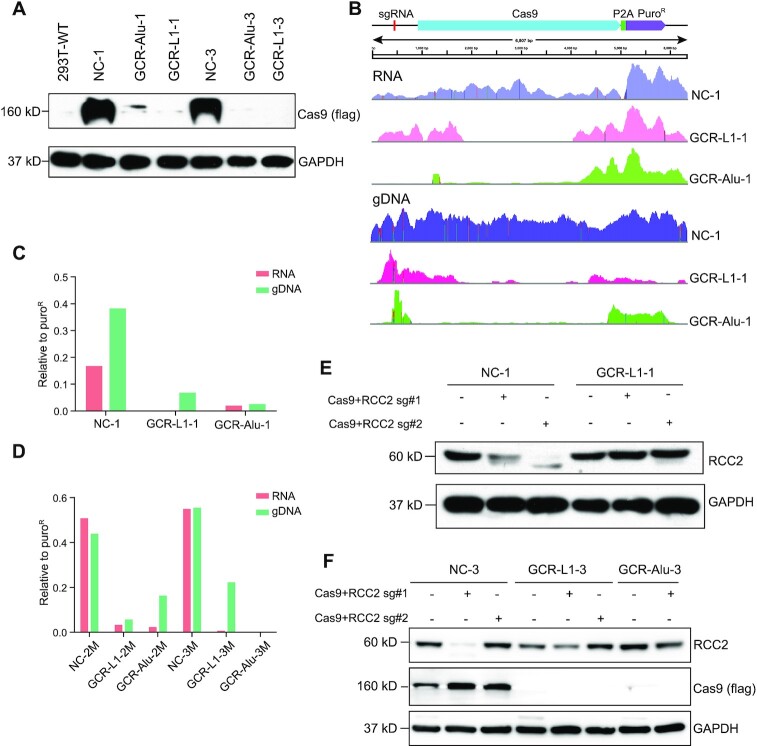
Cas9 was silenced in the GCR cells. (**A**) The expressions of Cas9 protein in NC and GCR cells. (**B**) The RNA-seq and WGS read coverages of the sgRNA-Cas9-Puro^R^ cassette. (**C**) The RNA-seq and WGS read coverage of the Cas9 (nt: 1123–3123) relative Puro^R^ in NC-1, GRC-L1-1 and GCR-Alu-1. (**D**) The RNA-seq and WGS read coverage of the Cas9 (nt: 1123–3123) relative Puro^R^ in the six single clones. (**E**) The expressions of RCC2 protein after NC-1 and GCR-L1-1 cells were transfected with pSB-CRISPR-Blast-sgRCC2 and selected by blasiticidin. (**F**) The expressions of RCC2 protein after NC-3, GCR-L1-3 and GCR-Alu-3 cells were transfected with pSB-CRISPR-Blast-sgRCC2 and selected by blasiticidin.

Cas9 and sgRNA expression cassettes were integrated into the host genomes and forced to express in the presence of puromycin. However, the growth and proliferation of the GCR cells after recovery was similar to that of the NC cells, without significant apoptosis observed, which indicated that Cas9 was no longer continuously cleaving the chromosomal DNAs. Theoretically, Cas9 or sgRNA, or both of them, should have been inhibited. Since the Cas9 version used here has a Flag-tag on the C-terminus (the same version as in lentiCRISPR v2 (61)), we detected the expression of Cas9 in NC and GCR cells from two independent transfections with anti-Flag antibody. Western blot showed that Cas9 proteins were almost undetectable in the GCR cells, whereas, the Cas9 proteins were highly expressed in the NC cells (Figure 7A). We checked the abundances of Cas9 mRNA in the RNA-seq data. Since Cas9 and puromycin resistance gene (Puro^R^) were from the same plasmid and integrated into the genome by SB100X transposase simultaneously, the expression of Puro^R^ is an ideal internal reference for the expression of Cas9. We compared the RNA sequencing reads of the three groups of cells and found that despite the high expressions of Puro^R^ in all of them, the Cas9 mRNA was only highly expressed in the NC cells. However, in both of the two GCR cells, the Cas9 mRNAs were no longer complete, with a loss of the central region (Figure 7B). According to the structure of Cas9 protein, the missing part mainly includes three domains, RecI, RuvC and HNH. RecI is the domain for sgRNA binding, while RuvC and HNH contain the endonuclease domain (62,63). The defect of these domains would certainly destroy the activity of Cas9.

We also checked the WGS data and found that the patterns of sequencing reads coverage were similar to those of RNA-seq (Figure 7B). It is not surprising that the cells that lost or partially lost Cas9 gene in the chromosome rearrangements dominated the cell pools. The quantitative comparison of the RNA expression of this region (nt: 1123–3123; aa: 375–1041) also showed significant drops in the two GCR cells (Figure 7C).

Then, the same analysis was performed for the single clones (Figure 7D). In brief, the Cas9/Puro^R^ ratios at RNA level dropped drastically in all the clones, but some clones still showed moderate values at genomic DNA level, such as GCR-Alu-2M and GCR-L1-3M. We also detected the mRNA expression of Cas9 and Puro^R^ using qRT-PCR and the result showed the similar trend as observed in the RNA-seq (Supplementary Figure S12). Probably, those cells also developed certain mechanisms to silence Cas9 at the RNA level.

We further transfected the GCR-L1-1 and NC-1 cells with the pSB-CRISPR-Blast plasmids containing Cas9 and sgRNAs. The knockout of RCC2 gene with pSB-CRISPR has been successfully performed previously (18), so it is a good positive control for testing whether the CRISPR-Cas9 system is working well. The western blot of Figure 7E showed that the RCC2 gene was successfully knocked out in NC-1 cells, but not in GCR-L1-1 cells. The assay was further repeated in NC-3, GCR-L1-3 and GCR-Alu-3 cells, and the result was similar (Figure 7F). Moreover, Cas9 protein was undetectable in the GCR cells, though they had been transfected with the pSB plasmids again. This result indicated that the GCR cells may silence the Cas9 endonuclease at both genomic DNA and mRNA levels to facilitate the cells survival.

Additionally, the Puro^R^ mRNA is in the same open reading frame with Cas9 mRNA, separated by P2A. After the central region of the mRNA was degraded, the translation of the 3′ part of the mRNA may start from one of the methionines at the Cas9 C-terminus, resulting in a Cas9 C-terminus fragment and an intact puromycin-N-acetyltransferase protein.

## DISCUSSION

**Figure 8.**
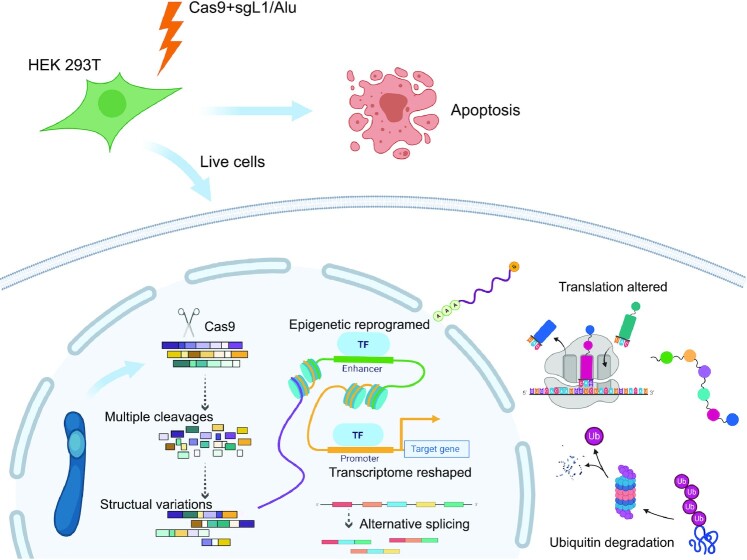
GCR brings great impact to cells. GCR is induced by targeting repetitive elements with CRISPR-Cas9. The 3D structure of the genome is changed; the distribution of nucleosomes becomes disorder; and the gene expression profile is reshaped; the peptide translation is altered and many mistake proteins are degraded by ubiquitin pathway.

CRISPR genome editing tool is a powerful technology. In recent years, new applications based on CRISPR are also emerging rapidly and continuously. In this study, we developed a method, CReaC, which causes global chromosome rearrangement in the human genome, and deeply reshapes the landscapes of epigenetics and gene expression of the cells (Figure 8). The wine yeast (*S. cerevisiae*) has a genome of only ∼12 Mb, while the mammalian genomes are much larger and more complex, e.g. the haploid human and mouse genomes are ∼3 Gb, so that it is impossible to generate GCR by synthesizing chromosomes at the current stage like the operation in yeast. CReaC provides an approach to study chromosome rearrangement in mammalian cells as well as in other complex eukaryotic cells.

In most cases, people use CRISPR-Cas9 to edit a specific site (two sites in diploid genomes) accurately, and try to avoid off-targets. However, there were also examples of targeting multiple genomic loci with CRISPR-Cas9. Huimin Zhao's group used CRISPR-Cas9 to achieve multi-disruption and integration in *S. cerevisiae* (64,65). Yang and Church have knocked out 60 PERV genes from the porcine genome (66,67), and they also used base editor (BE) to edit tens of thousands of repetitive elements without making DSBs in both 293T cells and pluripotent stem cells (68), but whether cells can survive over hundreds, thousands or even more DSBs remains unknown. In this study, we used sgRNAs with up to hundreds of thousands of matching sites to make DNA breakpoints and observed the subsequent changes, which, to our knowledge, is the first attempt so far. Although there have been several studies using CRISPR-Cas9 to generate chromosome rearrangements, those were only individual rearrangement in stead of global rearrangement (69,70).

The formation of the aneuploidy in carcinogenesis is a long-term and gradual process, and is difficult to investigate. Our research simulated this process in a considerably short time, which more intuitively explained the role of chromosome rearrangements in promoting cell carcinogenesis. Although the development and homeostasis of multicellular organisms are delicately tuned, our results suggest that their unicellular life mode, like immortalized cells or tumor cells, can be robust enough to tolerate severe chromosome rearrangements. The CNVs produced by chromosome rearrangements can only partially explain the changes in gene expression, and more changes were caused by gene expression regulations, in which epigenetics may play a key role. Our study showed that the distribution of nucleosomes tended to be disorder in the GCR cells. But it is unclear why the prime sequence changes in certain regions can cause the disorder distribution of nucleosomes globally.

It is interesting that GCR-Alu and GCR-L1 exhibit remarkably different, which might be due to that the copy number of Alu is far larger than that of L1 and the DNA DSBs in GCR-Alu are many more than those in GCR-L1, though the translocations and CNVs detected are similar. However, there is still other possibility that the cell status after GCR is not only determined by the chromosome rearrangement itself, but also is related to the functions of the elements where the DSBs take place. This result indicates that the physiological function of Alu might be even more important than L1 for its much higher copy number or for the transactivators interacting with them. Actually, recent studies showed that Alu elements are important for the formation of TADs and circRNAs (50,71,72). Although the genomic fraction of Alu is smaller than that of L1 (11% versus 17%), it might be a more important composition than L1.

In addition, other repetitive elements, such as satellite DNAs, microsatellite DNAs, telomeres, centromeres or rDNAs can also be target options for CReaC and CReaC could be an approach for studying the functions of various repetitive elements.

While large-scale chromosome rearrangements may also be generated by using chemical or physical stimuli that cause DNA DSBs, our method has obvious advantages: the target site sequences and the potential number of target sites can be designed according to requirements. Therefore, CReaC is more flexible and controllable than breaking DNA strands using chemical or physical reagents to generate random DSBs.

When exogenous Cas9 and sgRNA are introduced into eukaryotic cells, the cells will certainly have responses. Although CRISPR-Cas9 is a high-efficiency genome editing tool, it does exhibit low efficiency or even failure in many operations. It is reported that the causes of CRISPR failure are various and complicated (73). Here we observed that the host cells may silence the Cas9 expression at RNA level, especially the active center domain of Cas9 under certain circumstances, which provide a new angle to investigate the efficiency of CRISPR-editing. The study on the mechanism of the Cas9 silence may help promote or inhibit the activity of CRISPR-Cas9 in the future operations, and it can also improve the efficiency and safety of genome editing especially for therapeutic purpose.

Our current knowledge on the GCR cells is still limited, and all the findings of this study are based on immortalized cells. We are planning to perform CReaC in pluripotent stem cells in the future study. We are also interested in the 3D chromatin organization after GCR and may perform Hi-C assays to answer this question.

In summary, our study provided an easy-to-use and practical method for inducing GCR in mammalian cells and resource datasets of genomics, epigenomics and transcriptomics on the first GCR model in human cells.

## DATA AVAILABILITY

The edgeR (http://bioconductor.org/packages/release/bioc/html/edgeR.html), clusterProfiler (https://bioconductor.org/packages/release/bioc/html/clusterProfiler.html), DiffBind (https://bioconductor.org/packages/release/bioc/html/DiffBind.html) and ChIPseeker (https://bioconductor.org/packages/release/bioc/html/ChIPseeker.html) used for the analysis are available on Bioconductor. RCircos package (https://cran.r-project.org/web/packages/RCircos/index.html), pheatmap (https://cran.r-project.org/web/packages/pheatmap/index.html) and ggplot2 (https://cran.r-project.org/web/packages/ggplot2/index.html) are available via local R CRAN repository. Matplotlib package is a comprehensive library for visualizations in Python (https://matplotlib.org/). HISAT2 (http://www.ccb.jhu.edu/software/hisat/index.shtml), Bowtie2 (http://bowtiebio.sourceforge.net/%20Bowtie2%20/index.shtml) and BWA (https://github.com/lh3/bwa) were used as alignment programs. Bedtools was used for genome arithmetic (https://github.com/arq5x/bedtools2). CREST and Control-FREEC used for detecting genomic structural variations are available on Github (https://github.com/youngmook/CREST, https://github.com/BoevaLab/FREEC). BINGO (https://apps.cytoscape.org/apps/bingo) and CPDB (http://consensuspathdb.org/) calculated overrepresented GO terms and displayed them as networks. BioRender is a website for creating professional science figures (https://biorender.com/).

The raw sequencing data were deposited in the Sequence Read Archive (SRA, https://www.ncbi.nlm.nih.gov/sra/) under accession numbers PRJNA732565 (WGS data), PRJNA731018 (RNA-seq data), PRJNA731611 (ATAC-seq data) and PRJNA794061 (ONT sequencing) respectively.

## Supplementary Material

gkac153_Supplemental_Files

## References

[1] Richardson C. , Jasin M. Frequent chromosomal translocations induced by DNA double-strand breaks. Nature. 2000; 405:697–700. 10864328 10.1038/35015097

[2] Jacobs P.A. , Browne C., Gregson N., Joyce C., White H. Estimates of the frequency of chromosome abnormalities detectable in unselected newborns using moderate levels of banding. J. Med. Genet. 1992; 29:103–108. 1613759 10.1136/jmg.29.2.103 PMC1015848

[3] Petersen M.B. , Adelsberger P.A., Schinzel A.A., Binkert F., Hinkel G.K., Antonarakis S.E. Down syndrome due to de novo robertsonian translocation t(14q;21q): DNA polymorphism analysis suggests that the origin of the extra 21q is maternal. Am. J. Hum. Genet. 1991; 49:529–536. 1831959 PMC1683126

[4] Thompson S.L. , Compton D.A. Chromosomes and cancer cells. Chromosome Res. 2011; 19:433–444. 21190130 10.1007/s10577-010-9179-y PMC3770937

[5] Melo J.V. The molecular biology of chronic myeloid leukaemia. Leukemia. 1996; 10:751–756. 8656667

[6] Rieseberg L.H. Chromosomal rearrangements and speciation. Trends Ecol. Evol. 2001; 16:351–358. 11403867 10.1016/s0169-5347(01)02187-5

[7] Spielmann M. , Lupianez D.G., Mundlos S. Structural variation in the 3D genome. Nat. Rev. Genet. 2018; 19:453–467. 29692413 10.1038/s41576-018-0007-0

[8] Shao Y. , Lu N., Wu Z., Cai C., Wang S., Zhang L.L., Zhou F., Xiao S., Liu L., Zeng X. et al. Creating a functional single-chromosome yeast. Nature. 2018; 560:331–335. 30069045 10.1038/s41586-018-0382-x

[9] Shao Y. , Lu N., Cai C., Zhou F., Wang S., Zhao Z., Zhao G., Zhou J.Q., Xue X., Qin Z. A single circular chromosome yeast. Cell Res. 2019; 29:87–89. 30559437 10.1038/s41422-018-0110-y PMC6318310

[10] Shen Y. , Stracquadanio G., Wang Y., Yang K., Mitchell L.A., Xue Y., Cai Y., Chen T., Dymond J.S., Kang K. et al. SCRaMbLE generates designed combinatorial stochastic diversity in synthetic chromosomes. Genome Res. 2016; 26:36–49. 26566658 10.1101/gr.193433.115 PMC4691749

[11] Jones S. SCRaMbLE does the yeast genome shuffle. Nat. Biotechnol. 2018; 36:503. 29874209 10.1038/nbt.4164

[12] Luo Z. , Jiang S., Dai J. Chromosomal rearrangements of synthetic yeast by SCRaMbLE. Methods Mol. Biol. 2021; 2196:153–165. 32889719 10.1007/978-1-0716-0868-5_12

[13] Mercy G. , Mozziconacci J., Scolari V.F., Yang K., Zhao G., Thierry A., Luo Y., Mitchell L.A., Shen M., Shen Y. et al. 3D organization of synthetic and scrambled chromosomes. Science. 2017; 355:eaaf4597. 28280150 10.1126/science.aaf4597 PMC5679085

[14] Cong L. , Ran F.A., Cox D., Lin S., Barretto R., Habib N., Hsu P.D., Wu X., Jiang W., Marraffini L.A. et al. Multiplex genome engineering using CRISPR/Cas systems. Science. 2013; 339:819–823. 23287718 10.1126/science.1231143 PMC3795411

[15] Shalem O. , Sanjana N.E., Hartenian E., Shi X., Scott D.A., Mikkelson T., Heckl D., Ebert B.L., Root D.E., Doench J.G. et al. Genome-scale CRISPR-Cas9 knockout screening in human cells. Science. 2014; 343:84–87. 24336571 10.1126/science.1247005 PMC4089965

[16] Cordaux R. , Batzer M.A. The impact of retrotransposons on human genome evolution. Nat. Rev. Genet. 2009; 10:691–703. 19763152 10.1038/nrg2640 PMC2884099

[17] Langmead B. , Salzberg S.L. Fast gapped-read alignment with bowtie 2. Nat. Methods. 2012; 9:357–359. 22388286 10.1038/nmeth.1923 PMC3322381

[18] Hu K. , Li Y., Wu W., Chen H., Chen Z., Zhang Y., Guo Y., Dong Y. High-performance gene expression and knockout tools using sleeping beauty transposon system. Mobile DNA. 2018; 9:33. 30534207 10.1186/s13100-018-0139-y PMC6260868

[19] Zhang Y. , Huang Y.X., Wang D.L., Yang B., Yan H.Y., Lin L.H., Li Y., Chen J., Xie L.M., Huang Y.S. et al. LncRNA DSCAM-AS1 interacts with YBX1 to promote cancer progression by forming a positive feedback loop that activates FOXA1 transcription network. Theranostics. 2020; 10:10823–10837. 32929382 10.7150/thno.47830 PMC7482804

[20] Li H. , Durbin R. Fast and accurate short read alignment with burrows-wheeler transform. Bioinformatics. 2009; 25:1754–1760. 19451168 10.1093/bioinformatics/btp324 PMC2705234

[21] Wang J. , Mullighan C.G., Easton J., Roberts S., Heatley S.L., Ma J., Rusch M.C., Chen K., Harris C.C., Ding L. et al. CREST maps somatic structural variation in cancer genomes with base-pair resolution. Nat. Methods. 2011; 8:652–654. 21666668 10.1038/nmeth.1628 PMC3527068

[22] Boeva V. , Popova T., Bleakley K., Chiche P., Cappo J., Schleiermacher G., Janoueix-Lerosey I., Delattre O., Barillot E. Control-FREEC: a tool for assessing copy number and allelic content using next-generation sequencing data. Bioinformatics. 2012; 28:423–425. 22155870 10.1093/bioinformatics/btr670 PMC3268243

[23] Parkhomchuk D. , Borodina T., Amstislavskiy V., Banaru M., Hallen L., Krobitsch S., Lehrach H., Soldatov A. Transcriptome analysis by strand-specific sequencing of complementary DNA. Nucleic Acids Res. 2009; 37:e123. 19620212 10.1093/nar/gkp596 PMC2764448

[24] Kim D. , Langmead B., Salzberg S.L. HISAT: a fast spliced aligner with low memory requirements. Nat. Methods. 2015; 12:357–360. 25751142 10.1038/nmeth.3317 PMC4655817

[25] Memczak S. , Jens M., Elefsinioti A., Torti F., Krueger J., Rybak A., Maier L., Mackowiak S.D., Gregersen L.H., Munschauer M. et al. Circular RNAs are a large class of animal RNAs with regulatory potency. Nature. 2013; 495:333–338. 23446348 10.1038/nature11928

[26] Gao Y. , Wang J., Zhao F. CIRI: an efficient and unbiased algorithm for de novo circular RNA identification. Genome Biol. 2015; 16:4. 25583365 10.1186/s13059-014-0571-3 PMC4316645

[27] Buenrostro J.D. , Giresi P.G., Zaba L.C., Chang H.Y., Greenleaf W.J. Transposition of native chromatin for fast and sensitive epigenomic profiling of open chromatin, DNA-binding proteins and nucleosome position. Nat. Methods. 2013; 10:1213–1218. 24097267 10.1038/nmeth.2688 PMC3959825

[28] Zhang Y. , Liu T., Meyer C.A., Eeckhoute J., Johnson D.S., Bernstein B.E., Nusbaum C., Myers R.M., Brown M., Li W. et al. Model-based analysis of chip-Seq (MACS). Genome Biol. 2008; 9:R137. 18798982 10.1186/gb-2008-9-9-r137 PMC2592715

[29] Yu G. , Wang L.G., He Q.Y. ChIPseeker: an R/Bioconductor package for ChIP peak annotation, comparison and visualization. Bioinformatics. 2015; 31:2382–2383. 25765347 10.1093/bioinformatics/btv145

[30] De Coster W. , D’Hert S., Schultz D.T., Cruts M., Van Broeckhoven C. NanoPack: visualizing and processing long-read sequencing data. Bioinformatics. 2018; 34:2666–2669. 29547981 10.1093/bioinformatics/bty149 PMC6061794

[31] Li H. Minimap2: pairwise alignment for nucleotide sequences. Bioinformatics. 2018; 34:3094–3100. 29750242 10.1093/bioinformatics/bty191 PMC6137996

[32] Maere S. , Heymans K., Kuiper M. BiNGO: a cytoscape plugin to assess overrepresentation of gene ontology categories in biological networks. Bioinformatics. 2005; 21:3448–3449. 15972284 10.1093/bioinformatics/bti551

[33] Mootha V.K. , Lindgren C.M., Eriksson K.F., Subramanian A., Sihag S., Lehar J., Puigserver P., Carlsson E., Ridderstrale M., Laurila E. et al. PGC-1alpha-responsive genes involved in oxidative phosphorylation are coordinately downregulated in human diabetes. Nat Genet. 2003; 34:267–273. 12808457 10.1038/ng1180

[34] Subramanian A. , Tamayo P., Mootha V.K., Mukherjee S., Ebert B.L., Gillette M.A., Paulovich A., Pomeroy S.L., Golub T.R., Lander E.S. et al. Gene set enrichment analysis: a knowledge-based approach for interpreting genome-wide expression profiles. Proc. Nat. Acad. Sci. U.S.A. 2005; 102:15545–15550. 10.1073/pnas.0506580102 PMC1239896 16199517

[35] Bader G.D. , Hogue C.W. An automated method for finding molecular complexes in large protein interaction networks. BMC Bioinf. 2003; 4:2. 10.1186/1471-2105-4-2 PMC149346 12525261

[36] Mátés L. , Chuah M.K.L., Belay E., Jerchow B., Manoj N., Acosta-Sanchez A., Grzela D.P., Schmitt A., Becker K., Matrai J. et al. Molecular evolution of a novel hyperactive sleeping beauty transposase enables robust stable gene transfer in vertebrates. Nat. Genet. 2009; 41:753–761. 19412179 10.1038/ng.343

[37] Binz R.L. , Tian E., Sadhukhan R., Zhou D., Hauer-Jensen M., Pathak R. Identification of novel breakpoints for locus- and region-specific translocations in 293 cells by molecular cytogenetics before and after irradiation. Sci. Rep. 2019; 9:10554. 31332273 10.1038/s41598-019-47002-0 PMC6646394

[38] Maass P.G. , Barutcu A.R., Rinn J.L. Interchromosomal interactions: a genomic love story of kissing chromosomes. J. Cell Biol. 2019; 218:27–38. 30181316 10.1083/jcb.201806052 PMC6314556

[39] Krug U. , Ganser A., Koeffler H.P. Tumor suppressor genes in normal and malignant hematopoiesis. Oncogene. 2002; 21:3475–3495. 12032783 10.1038/sj.onc.1205322

[40] Serrano M. The tumor suppressor protein p16INK4a. Exp. Cell. Res. 1997; 237:7–13. 9417860 10.1006/excr.1997.3824

[41] Shapiro G.I. , Edwards C.D., Ewen M.E., Rollins B.J. p16INK4A participates in a G1 arrest checkpoint in response to DNA damage. Mol. Cell. Biol. 1998; 18:378–387. 9418885 10.1128/mcb.18.1.378 PMC121508

[42] Lee K.Y. , Dutta A. Chk1 promotes non-homologous end joining in G1 through direct phosphorylation of ASF1A. Cell Rep. 2021; 34:108680. 33503415 10.1016/j.celrep.2020.108680 PMC7941734

[43] Vafa O. , Wade M., Kern S., Beeche M., Pandita T.K., Hampton G.M., Wahl G.M. c-Myc can induce DNA damage, increase reactive oxygen species, and mitigate p53 function: a mechanism for oncogene-induced genetic instability. Mol Cell. 2002; 9:1031–1044. 12049739 10.1016/s1097-2765(02)00520-8

[44] Belancio V.P. , Hedges D.J., Deininger P. LINE-1 RNA splicing and influences on mammalian gene expression. Nucleic Acids Res. 2006; 34:1512–1521. 16554555 10.1093/nar/gkl027 PMC1415225

[45] Payer L.M. , Steranka J.P., Ardeljan D., Walker J., Fitzgerald K.C., Calabresi P.A., Cooper T.A., Burns K.H. Alu insertion variants alter mRNA splicing. Nucleic Acids Res. 2019; 47:421–431. 30418605 10.1093/nar/gky1086 PMC6326789

[46] Gal-Mark N. , Schwartz S., Ast G. Alternative splicing of alu exons–two arms are better than one. Nucleic Acids Res. 2008; 36:2012–2023. 18276646 10.1093/nar/gkn024 PMC2330237

[47] Lee J.R. , Park S.J., Kim Y.H., Choe S.H., Cho H.M., Lee S.R., Kim S.U., Kim J.S., Sim B.W., Song B.S. et al. Alu-Derived alternative splicing events specific to macaca lineages in CTSF gene. Mol. Cells. 2017; 40:100–108. 28196413 10.14348/molcells.2017.2204 PMC5339500

[48] Kornblihtt A.R. , Schor I.E., Allo M., Dujardin G., Petrillo E., Munoz M.J. Alternative splicing: a pivotal step between eukaryotic transcription and translation. Nat. Rev. Mol. Cell Biol. 2013; 14:153–165. 23385723 10.1038/nrm3525

[49] Zhang Y. , Qian J., Gu C., Yang Y. Alternative splicing and cancer: a systematic review. Signal Transduct. Targeted Ther. 2021; 6:78. 10.1038/s41392-021-00486-7 PMC7902610 33623018

[50] Dong R. , Ma X.K., Chen L.L., Yang L. Increased complexity of circRNA expression during species evolution. RNA Biol. 2017; 14:1064–1074. 27982734 10.1080/15476286.2016.1269999 PMC5680680

[51] Chen L.L. The biogenesis and emerging roles of circular RNAs. Nat. Rev. Mol. Cell Biol. 2016; 17:205–211. 26908011 10.1038/nrm.2015.32

[52] Casimiro M.C. , Velasco-Velazquez M., Aguirre-Alvarado C., Pestell R.G. Overview of cyclins D1 function in cancer and the CDK inhibitor landscape: past and present. Expert Opin. Investig. Drugs. 2014; 23:295–304. 10.1517/13543784.2014.867017 24387133

[53] Ihry R.J. , Worringer K.A., Salick M.R., Frias E., Ho D., Theriault K., Kommineni S., Chen J., Sondey M., Ye C. et al. p53 inhibits CRISPR-Cas9 engineering in human pluripotent stem cells. Nat Med. 2018; 24:939–946. 29892062 10.1038/s41591-018-0050-6

[54] Surget S. , Khoury M.P., Bourdon J.C. Uncovering the role of p53 splice variants in human malignancy: a clinical perspective. OncoTargets Ther. 2013; 7:57–68. 10.2147/OTT.S53876 PMC3872270 24379683

[55] Solomon H. , Sharon M., Rotter V. Modulation of alternative splicing contributes to cancer development: focusing on p53 isoforms, p53beta and p53gamma. Cell Death Differ. 2014; 21:1347–1349. 25109594 10.1038/cdd.2014.99 PMC4131187

[56] Ghosh A. , Stewart D., Matlashewski G. Regulation of human p53 activity and cell localization by alternative splicing. Mol. Cell. Biol. 2004; 24:7987–7997. 15340061 10.1128/MCB.24.18.7987-7997.2004 PMC515058

[57] Chen J. , Crutchley J., Zhang D., Owzar K., Kastan M.B. Identification of a DNA damage-induced alternative splicing pathway that regulates p53 and cellular senescence markers. Cancer Discov. 2017; 7:766–781. 28288992 10.1158/2159-8290.CD-16-0908 PMC5501752

[58] Ong C.T. , Corces V.G. CTCF: an architectural protein bridging genome topology and function. Nat. Rev. Genet. 2014; 15:234–246. 24614316 10.1038/nrg3663 PMC4610363

[59] Lu J.Y. , Chang L., Li T., Wang T., Yin Y., Zhan G., Han X., Zhang K., Tao Y., Percharde M. et al. Homotypic clustering of L1 and B1/Alu repeats compartmentalizes the 3D genome. Cell Res. 2021; 31:613–630. 33514913 10.1038/s41422-020-00466-6 PMC8169921

[60] Conti A. , Carnevali D., Bollati V., Fustinoni S., Pellegrini M., Dieci G. Identification of RNA polymerase III-transcribed alu loci by computational screening of RNA-Seq data. Nucleic Acids Res. 2015; 43:817–835. 25550429 10.1093/nar/gku1361 PMC4333407

[61] Sanjana N.E. , Shalem O., Zhang F. Improved vectors and genome-wide libraries for CRISPR screening. Nat. Methods. 2014; 11:783–784. 25075903 10.1038/nmeth.3047 PMC4486245

[62] Jinek M. , Jiang F., Taylor D.W., Sternberg S.H., Kaya E., Ma E., Anders C., Hauer M., Zhou K., Lin S. et al. Structures of cas9 endonucleases reveal RNA-mediated conformational activation. Science. 2014; 343:1247997. 24505130 10.1126/science.1247997 PMC4184034

[63] Nishimasu H. , Ran F.A., Hsu P.D., Konermann S., Shehata S.I., Dohmae N., Ishitani R., Zhang F., Nureki O. Crystal structure of cas9 in complex with guide RNA and target DNA. Cell. 2014; 156:935–949. 24529477 10.1016/j.cell.2014.02.001 PMC4139937

[64] Bao Z. , Xiao H., Liang J., Zhang L., Xiong X., Sun N., Si T., Zhao H. Homology-integrated CRISPR-Cas (HI-CRISPR) system for one-step multigene disruption in saccharomyces cerevisiae. ACS Synth. Biol. 2015; 4:585–594. 25207793 10.1021/sb500255k

[65] Shi S. , Liang Y., Zhang M.M., Ang E.L., Zhao H. A highly efficient single-step, markerless strategy for multi-copy chromosomal integration of large biochemical pathways in saccharomyces cerevisiae. Metab. Eng. 2016; 33:19–27. 26546089 10.1016/j.ymben.2015.10.011

[66] Yang L. , Guell M., Niu D., George H., Lesha E., Grishin D., Aach J., Shrock E., Xu W., Poci J. et al. Genome-wide inactivation of porcine endogenous retroviruses (PERVs). Science. 2015; 350:1101–1104. 26456528 10.1126/science.aad1191

[67] Niu D. , Wei H.-J., Lin L., George H., Wang T., Lee I.H., Zhao H.-Y., Wang Y., Kan Y., Shrock E. et al. Inactivation of porcine endogenous retrovirus in pigs using CRISPR-Cas9. Science. 2017; 357:1303–1307. 28798043 10.1126/science.aan4187 PMC5813284

[68] Smith C.J. , Castanon O., Said K., Volf V., Khoshakhlagh P., Hornick A., Ferreira R., Wu C.T., Guell M., Garg S. et al. Enabling large-scale genome editing at repetitive elements by reducing DNA nicking. Nucleic Acids Res. 2020; 48:5183–5195. 32315033 10.1093/nar/gkaa239 PMC7229841

[69] Vanoli F. , Tomishima M., Feng W., Lamribet K., Babin L., Brunet E., Jasin M. CRISPR-Cas9-guided oncogenic chromosomal translocations with conditional fusion protein expression in human mesenchymal cells. Proc. Nat. Acad. Sci. U.S.A. 2017; 114:3696–3701. 10.1073/pnas.1700622114 PMC5389291 28325870

[70] Reimer J. , Knoss S., Labuhn M., Charpentier E.M., Gohring G., Schlegelberger B., Klusmann J.H., Heckl D CRISPR-Cas9-induced t(11;19)/MLL-ENL translocations initiate leukemia in human hematopoietic progenitor cells in vivo. Haematologica. 2017; 102:1558–1566. 28572162 10.3324/haematol.2017.164046 PMC5685230

[71] Gong Y. , Lazaris C., Sakellaropoulos T., Lozano A., Kambadur P., Ntziachristos P., Aifantis I., Tsirigos A. Stratification of TAD boundaries reveals preferential insulation of super-enhancers by strong boundaries. Nat. Commun. 2018; 9:542. 29416042 10.1038/s41467-018-03017-1 PMC5803259

[72] Chen X. , Ke Y., Wu K., Zhao H., Sun Y., Gao L., Liu Z., Zhang J., Tao W., Hou Z. et al. Key role for CTCF in establishing chromatin structure in human embryos. Nature. 2019; 576:306–310. 31801998 10.1038/s41586-019-1812-0

[73] Wang H. , La Russa M., Qi L.S. CRISPR/Cas9 in genome editing and beyond. Annu. Rev. Biochem. 2016; 85:227–264. 27145843 10.1146/annurev-biochem-060815-014607 PMC13384723

