# Supplementary material for: Global chromosome rearrangement induced by CRISPR-Cas9 reshapes the genome and transcriptome of human cells
Source: Nucleic Acids Res. 2022 Mar 4;50(6):3456–74. doi: 10.1093/nar/gkac153 (PMC8989517; doi:10.1093/nar/gkac153)

Supplementary Tables
(All tables are in separated Excel files)

Supplementary Table 1. The sgRNA candidates for targeting L1 and Alu, the sequences and numbers of sites in hg38 were shown.

Supplementary Table 2. The translocation/inversion events in GCR genomes.

Supplementary Table 3. The genes that are located in the high CNV regions of GCR vs NC cells and their frequencies found in the clinical tumor samples of Cosmic.

Supplementary Table 4. The differentially expressed genes (DEGs) of GCR vs NC cells, including mRNAs and lncRNAs at gene and transcript levels.

Supplementary Table 5. The alternative splicing (AS) of GCR cells relative to NC.

Supplementary Table 6. The expression of circRNAs.

Supplementary Table 7. The peak analysis of ATAC-seq, the sequencing data were normalized by the total matches against the nuclear genome.

Supplementary Figure Legends

Supplementary Fig. 1.
A, the match sites of the sgRNAs used in this study. The number of exact matches, matches with 1 mismatch and matched with 2 mismatch were searched using bowtie2. B, the experiment scheme of CReaC and colonization. C, the growth rates of single clone cells with the wild type 293T cell.

Supplementary Fig. 2.
The translocations/inversions and the CNVs of the single clones. A, GCR-L1-2M vs NC-2M; B, GCR-Alu-2M vs NC-2M; C, GCR-L1-3M vs NC-3M; D, GCR-Alu-3M vs NC-3M.

Supplementary Fig. 3.
The comparison between inter-chromosomal and intra-chromosomal (including inversions). A, the numbers of intra- and inter-chromosomal translocations were

compared to the Monte Carlo (MC) simulations (see Methods for the MC simulation). The P-values were determined by Chi-square test (****, $P < 0.0001$). B and C, 2D plots show the intra- and inter-chromosome translocations in the four single clones (GCR-L1-2M, GCR-Alu-2M, GCR-L1-3M and GCR-Alu-3M) (B) and the matched MC simulation (C).

Supplementary Fig. 4.
A, The distances of the translocation breakpoints to the nearest L1/Alu elements of GCR-L1-1 and GCR-Alu-1. B and C, examples of translocations in GCR-L1-3M (B) and GCR-Alu-3M (C) detected in ONT sequencing.

Supplementary Fig. 5.
The gain and loss frequencies in clinical tumor samples of the genes at the high CNV regions in the four single clones (as labeled on the charts) relative to the responding NC clones, The frequencies were calculated using TCGA clinical samples data obtained from Cosmic. Genes that have been reported to play important roles in multiple cancer types were indicated in red.

Supplementary Fig. 6
Global Transcriptome Profiling of NC and GCR cells. A & B, the H-cluster of expression of mRNAs (A) and lncRNAs (B) at transcript-level. C - F, volcano plots show the differential expressions of mRNA (C & D) and lncRNA (E & F) at transcript-level in GCR-L1-1 vs NC-1 (C & E) and GCR-Alu-1 vs NC-1 (D & F). G, the H-cluster of mRNA expression in six single clones. H, number of up-regulated and down-regulated DEGs when GCR cells were compared to NC cells. I, Pearson correlation between expression of cell pools in transcriptome sequencing. J, Pearson correlation between expressions of single clones in mRNA sequencing.

Supplementary Fig. 7
Differential analysis of alternative splicing (AS) and circRNA. A, the different types of differential AS in GCR-L1-1 and GCR-Alu-1 compared to NC-1. AS are classified into the following five types: skipped exon (SE), mutually exclusive exon (MXE), alternative 5' splice site (A5SS), alternative 3' splice site (A3SS) and retained intron (RI). B, violin plot shows distribution of circRNA expression levels in NC and GCR cells. C, H-cluster of circRNA expressions in NC and GCR cells. D & E, volcano plots show the differential expressions of circRNA in GCR-L1-1 vs NC-1 (D) and GCR-Alu-1 vs NC-1 (E). F, numbers of the significant differential circRNA in GCR-L1-1 vs NC-1 and GCR-Alu-1 vs NC-1. G & H, the KEGG enrichment analysis of host genes of significant differential circRNA GCR-L1-1 vs NC-1 (G) and

GCR-Alu-1 vs NC-1 (H).

Supplementary Fig. 8

The common expression changes between GCR-L1-1 and GCR-Alu-1. A, the correlation of expression changes at transcript level between GCR-L1-1 vs NC-1 and GCR-Alu-1 vs NC-1 (R^2=0.2059). B & C, the expression fold changes at transcript level and corresponding gene level in GCR-L1-1 vs NC-1 (B) and GCR-Alu-1 vs NC-1 (C). The red points indicate transcript-level fold change has at least 2-fold increase or 0.5-fold decrease versus corresponding gene-level fold change. D, the correlation of fold change between GCR-L1-1 and GCR-Alu-1 for the genes marked red in panel Supplementary Fig. 8B & 8C. The red points indicate the genes with same regulated trend in the two GCR cells and with $|log_2FC| \geqslant 1$. E, the number of genes that marked in panel Supplementary Fig. 8D. F, GO enrichment analysis of genes marked red in Supplementary Fig. 8D is shown as an interaction network using Cytoscape plug-in, BinGO. The hierarchical relationships of GO terms are connected by arrows. Terms with $p<0.05$ are colored. G, the 192 genes marked red in Fig. 5A were analyzed using WikiPathway and Reactome. H, the 520 genes marked red in Supplementary Fig. 8D were analyzed using WikiPathway and Reactome.

Supplementary Fig. 9

Multiple pathways associated to cell survival were altered in GCR single clones. A, heatmap of enriched terms across four sets of DEGs, colored by p-values. B, for 135 common DEGs as shown in Fig 4I, the network of enriched terms are colored by clusters, where terms with a similarity > 0.3 are connected by edges. Terms with the best p-values from each of the clusters are shown as labels. C, MCODE components network identified across four sets of DEGs, carried out by PPI enrichment analysis and MCODE algorithm. The best-scoring terms by p-value are retained as the functional description of the corresponding components.

Supplementary Fig. 10

A-C, GSEA Enrichment plot: apoptosis (A), Myc targets v1 (B), Myc targets v2 (C).
D, the expression level of alternative splicing of TP53 gene.

Supplementary Fig. 11.

A, Principal component analysis (PCA) of ATAC-seq samples. PC1 and PC2 can explain 91.7% of the overall variance. B, Pearson correlation between ATAC-seq samples. C, distribution of distance to transcription start site (TSS) of common peaks in 3 samples. The abscissa indicates the percentage in different distances, and the ordinate indicates the sample.

Supplementary Fig. 12.

RT-qPCR assay shows the relative mRNA level of Cas9 and puromycin resistance gene in NC and GCR cells. A, cells pools; B, single clones. The P-values were determined by Student's t test (***, $P < 0.001$).

Supplementary Fig. 1

A

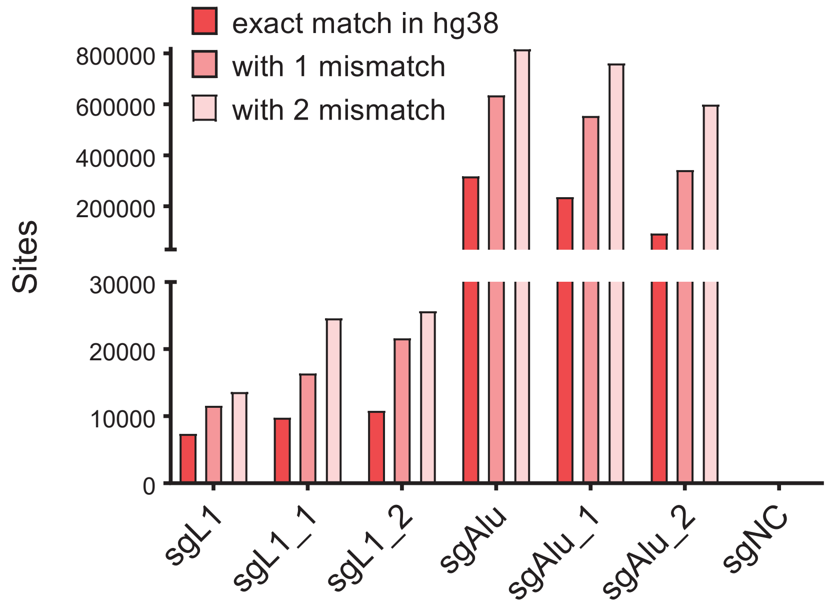

B

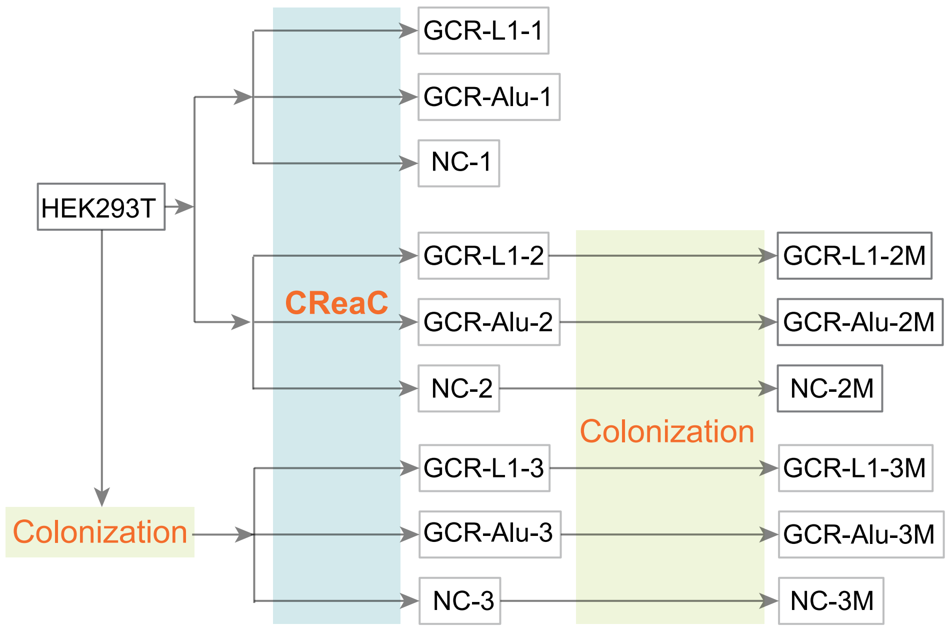

C

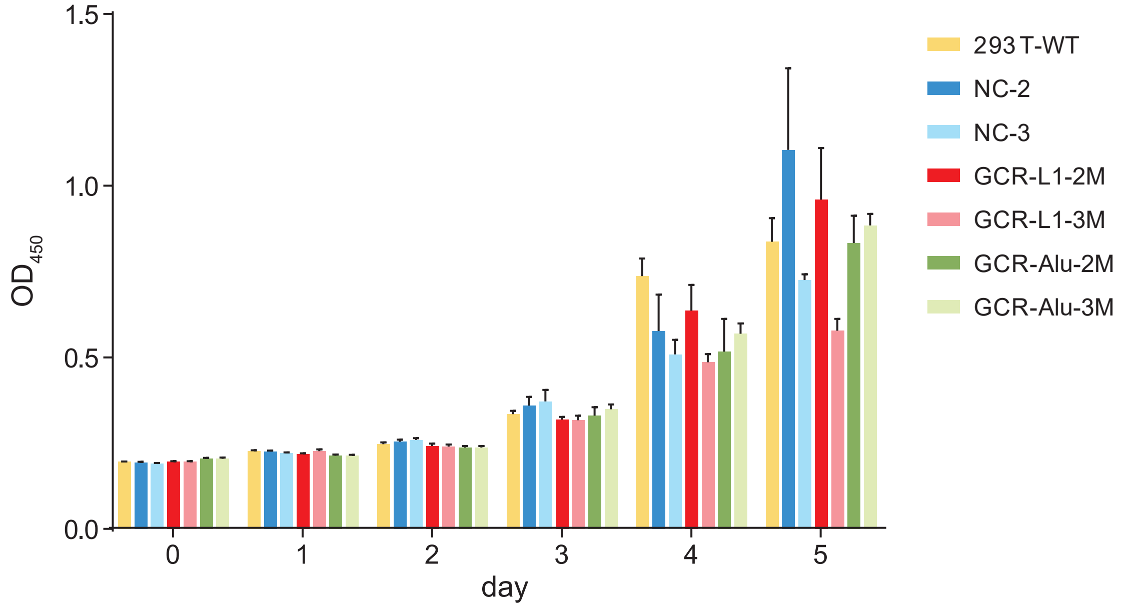

Supplementary Fig. 2

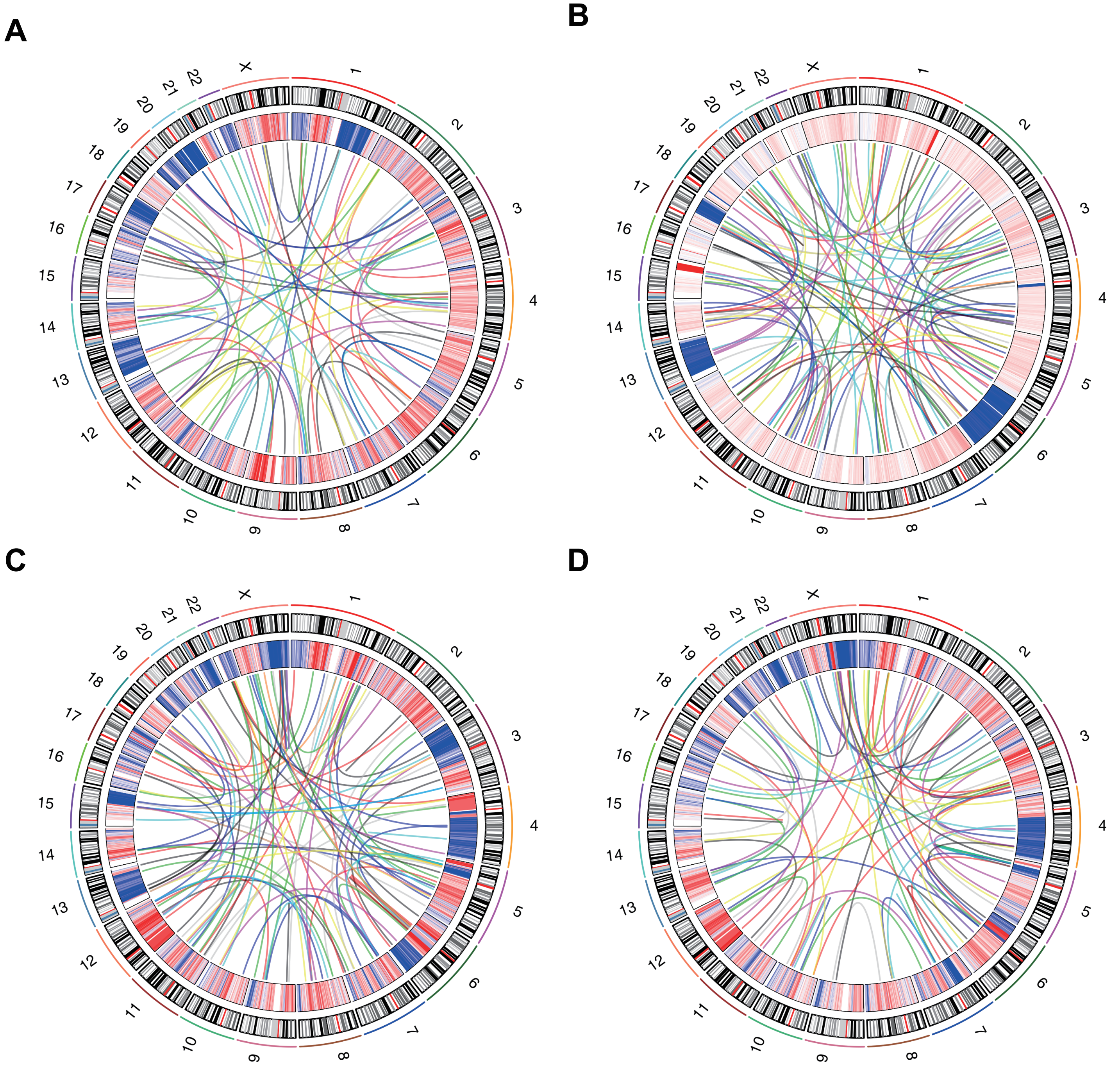

Supplementary Fig. 3

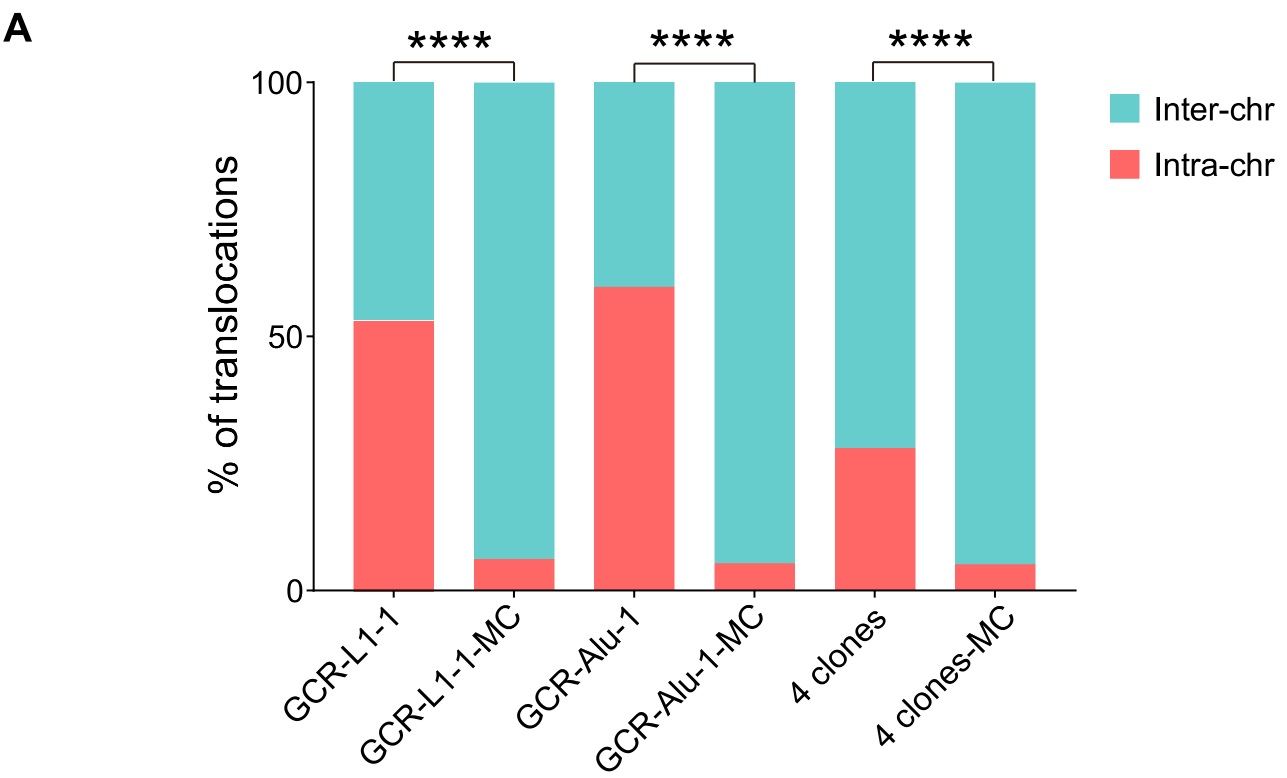

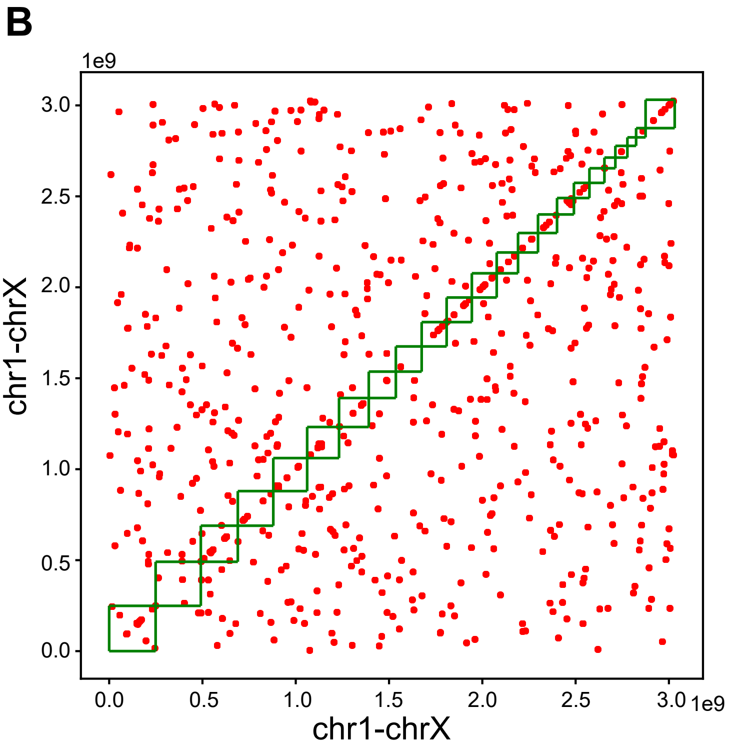

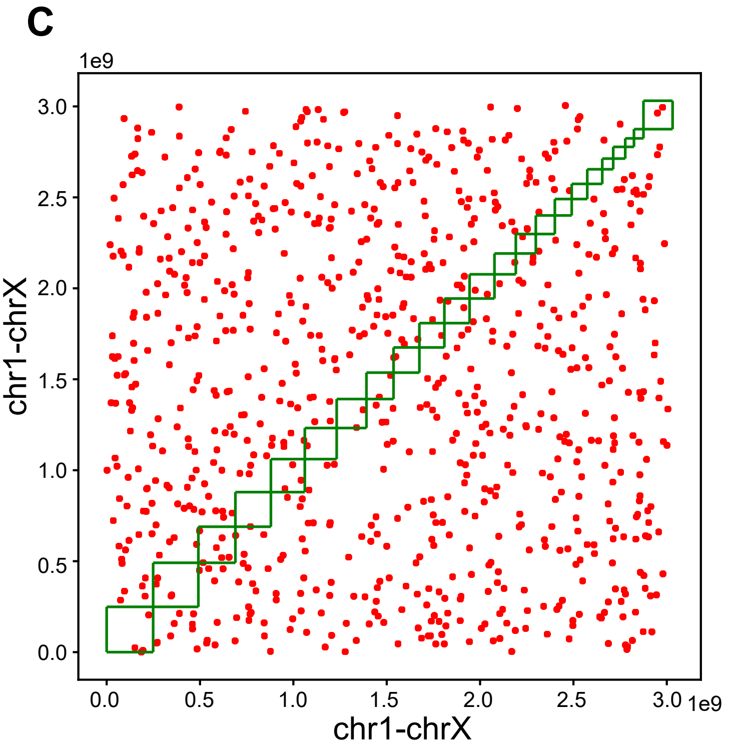

Supplementary Fig. 4

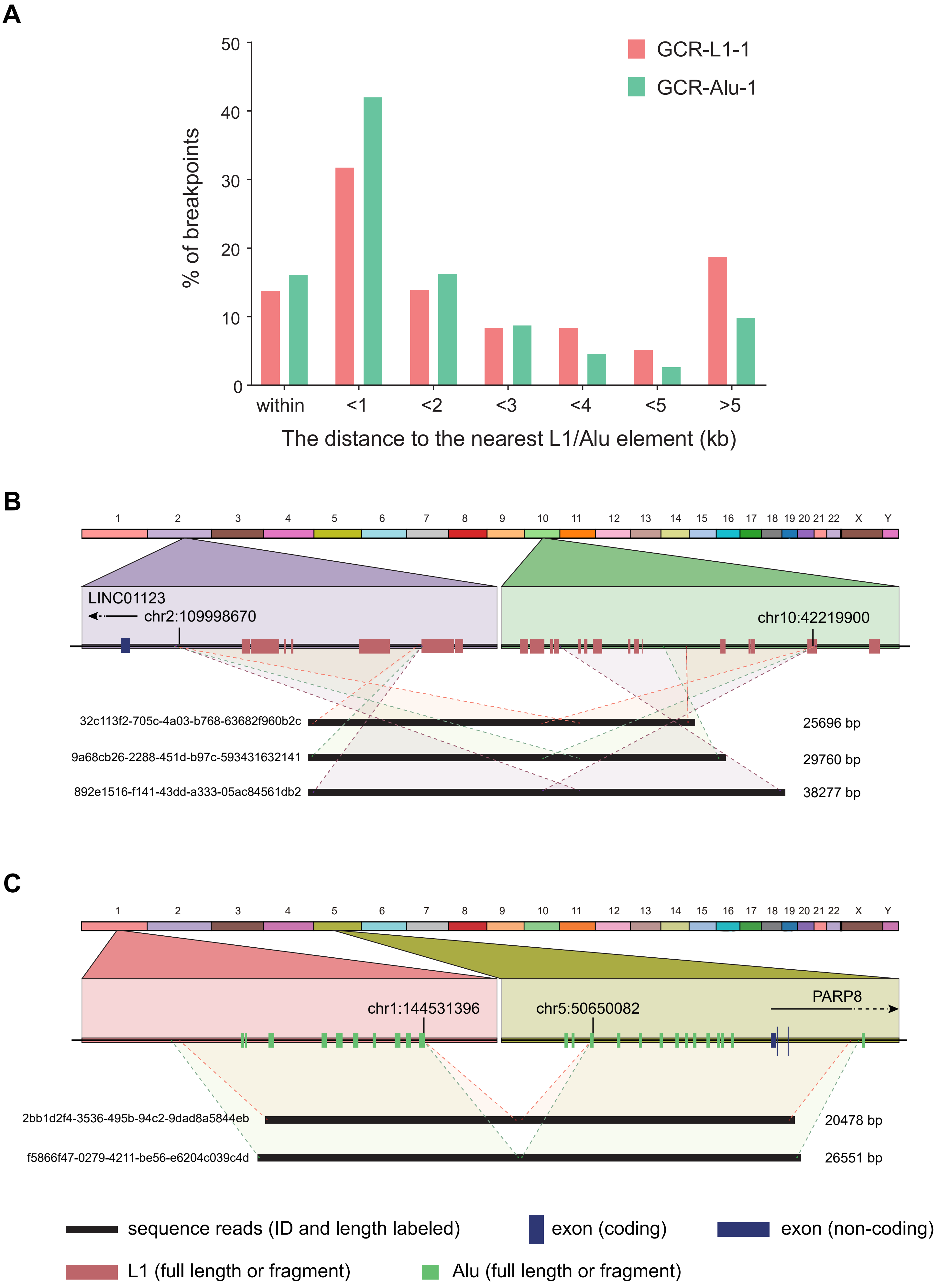

Supplementary Fig. 5

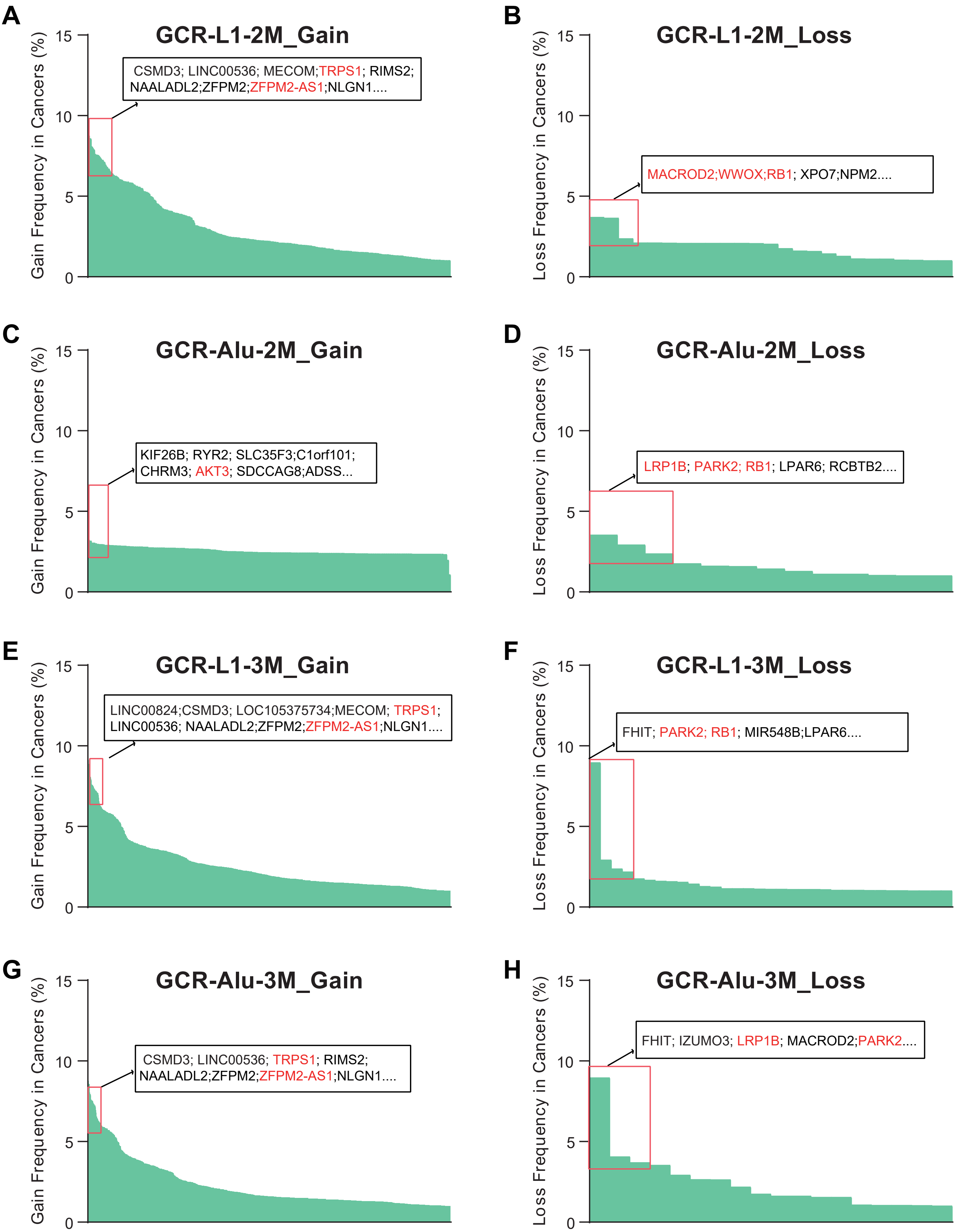

Supplementary Fig. 6

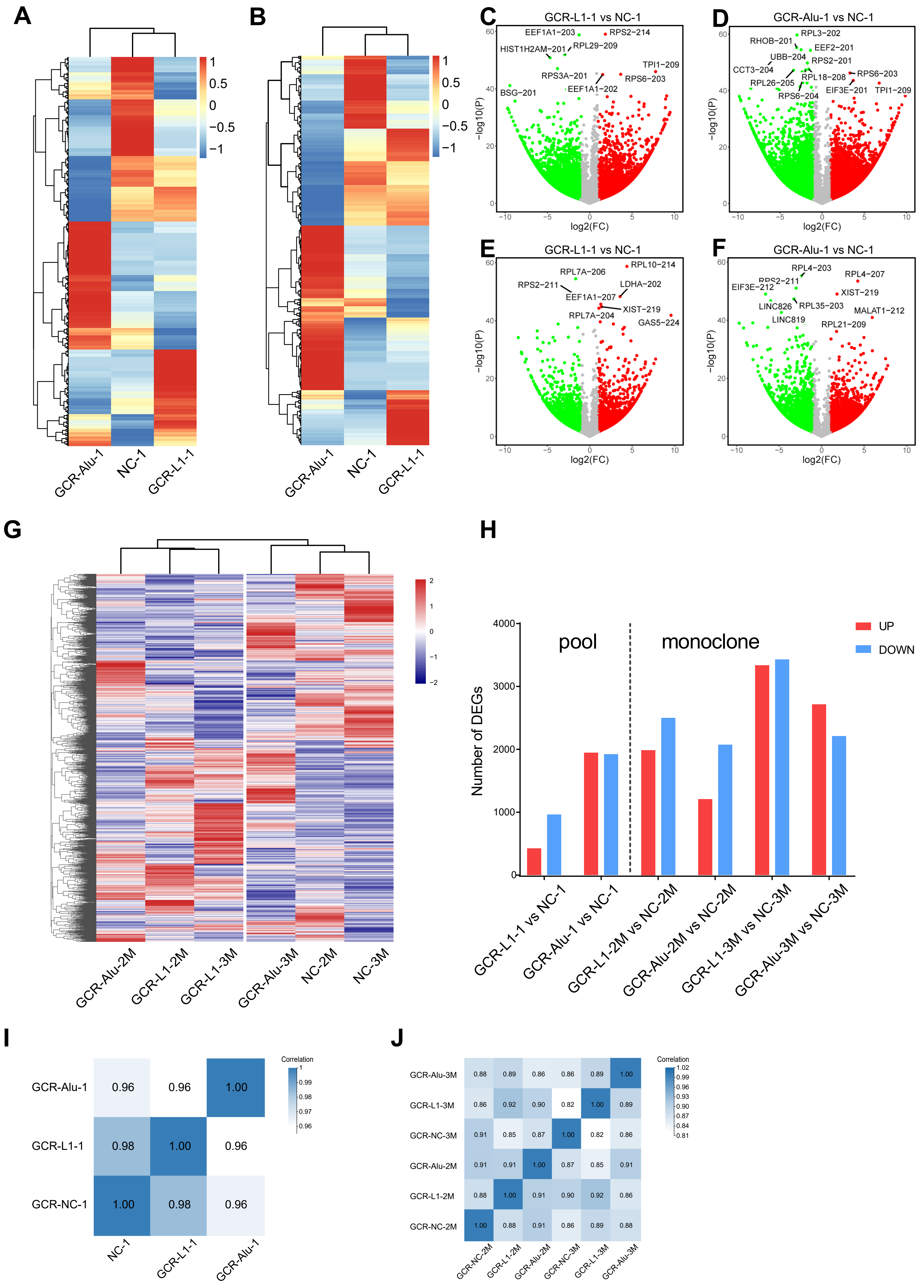

Supplementary Fig. 7

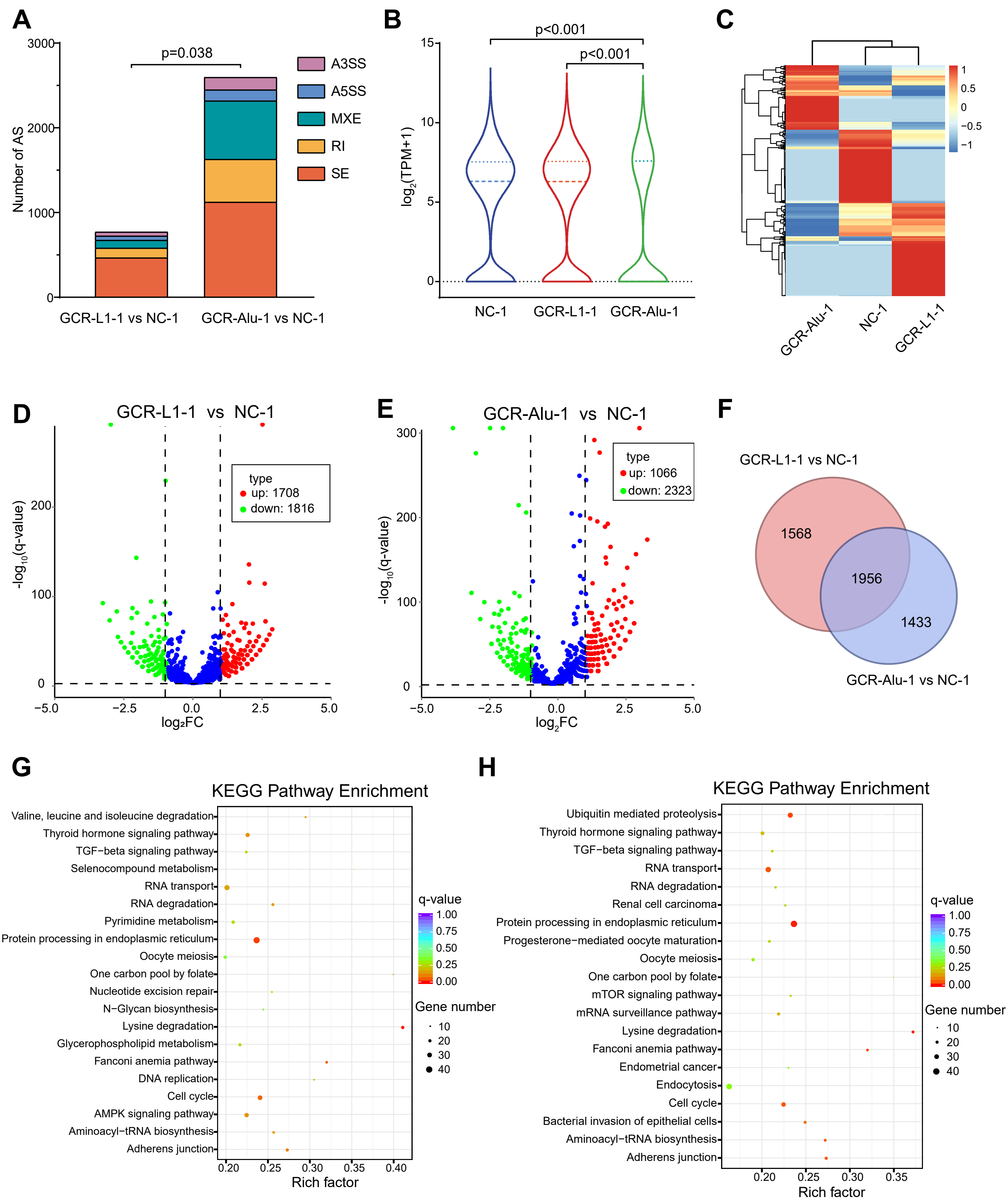

Supplementary Fig. 8

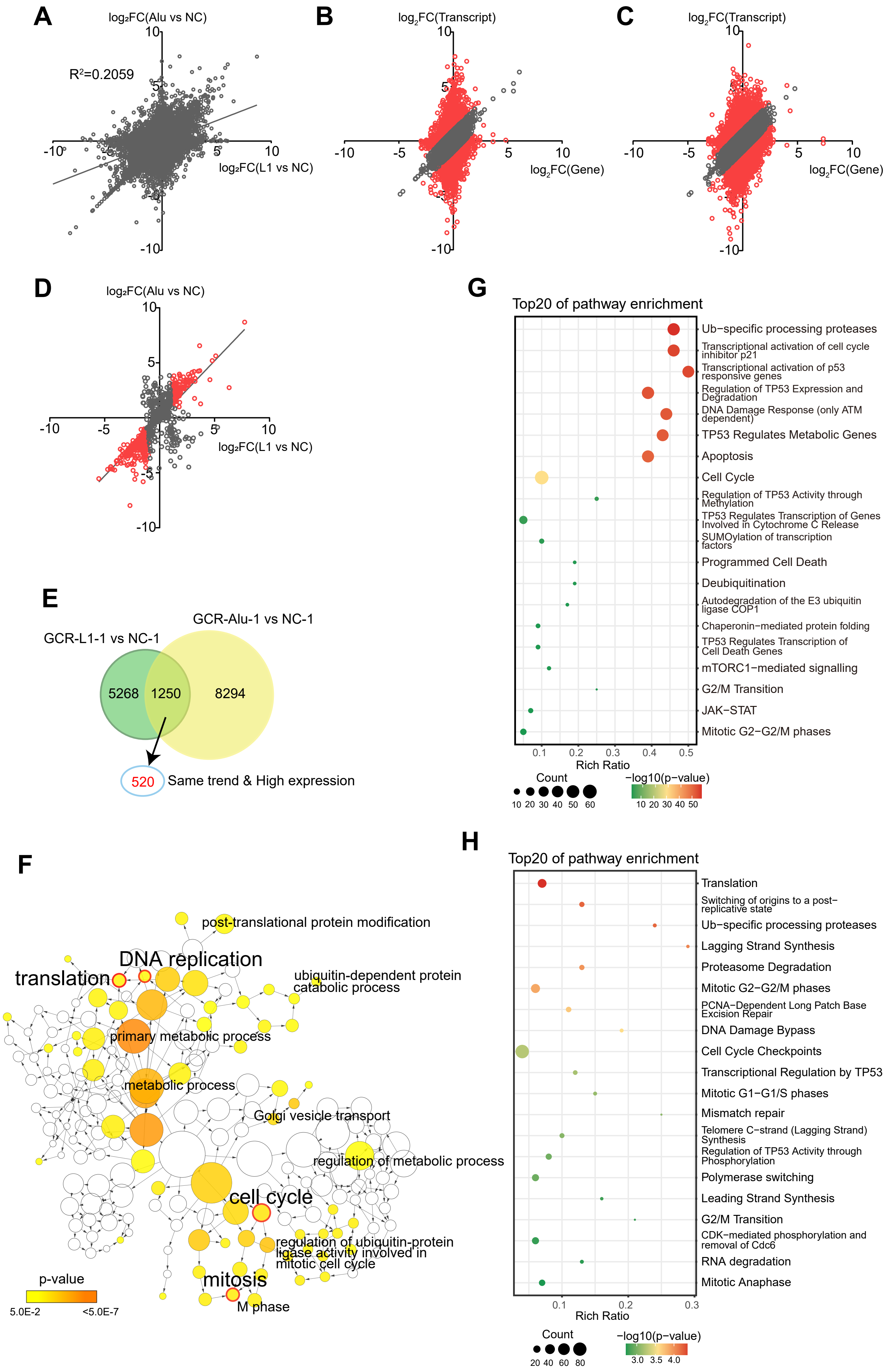

Supplementary Fig. 9

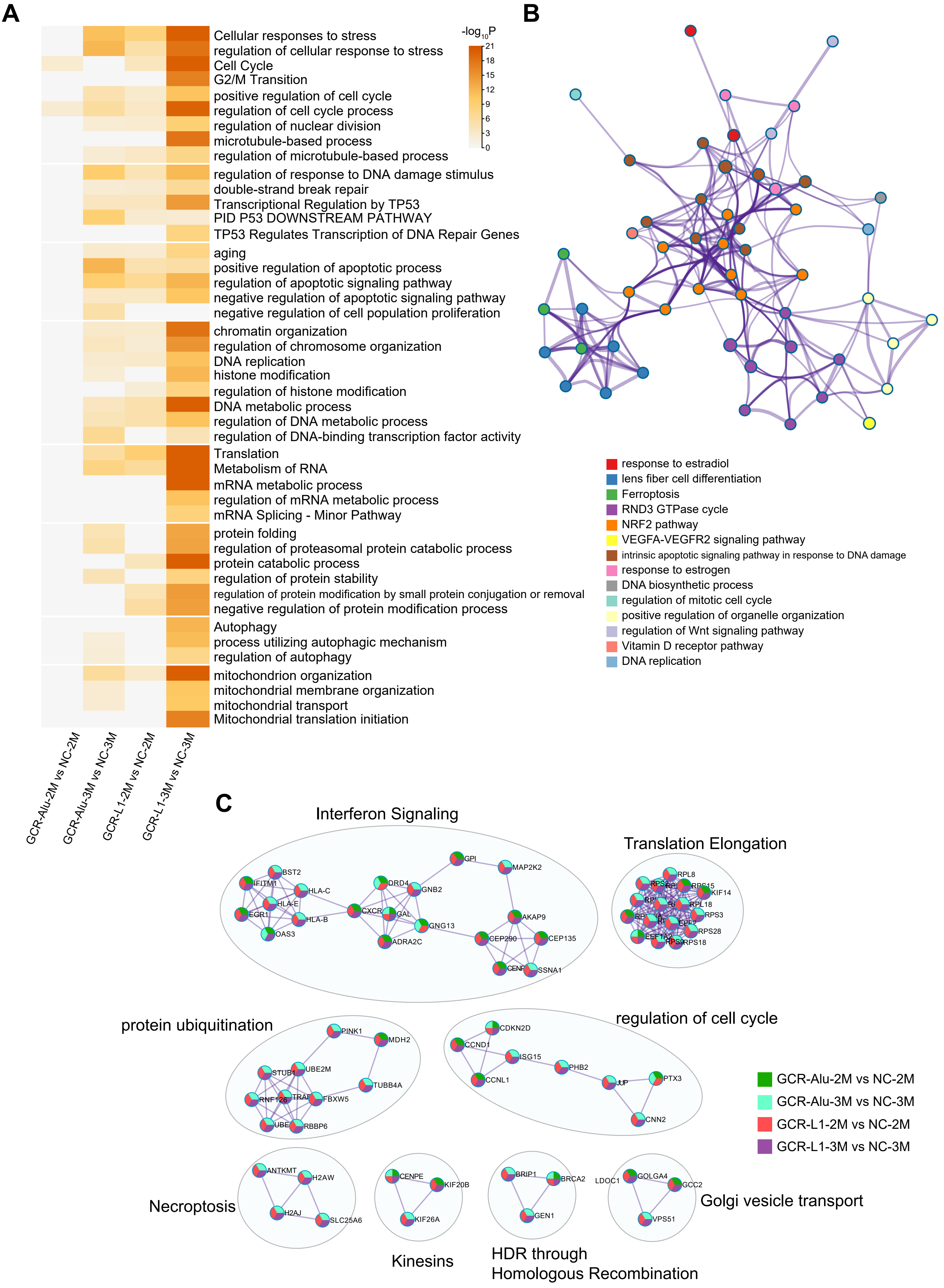

Supplementary Fig. 10

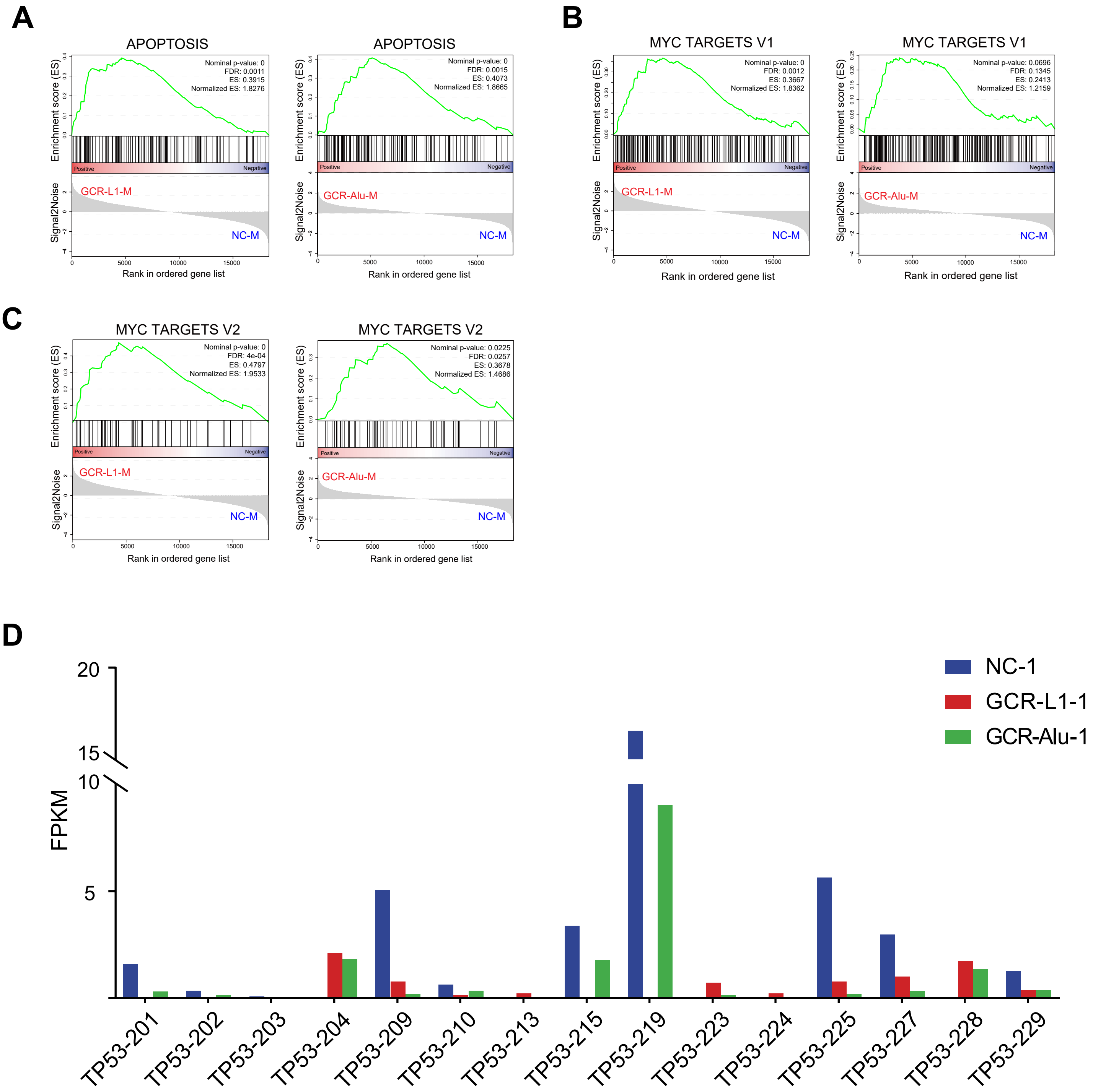

Supplementary Fig. 11

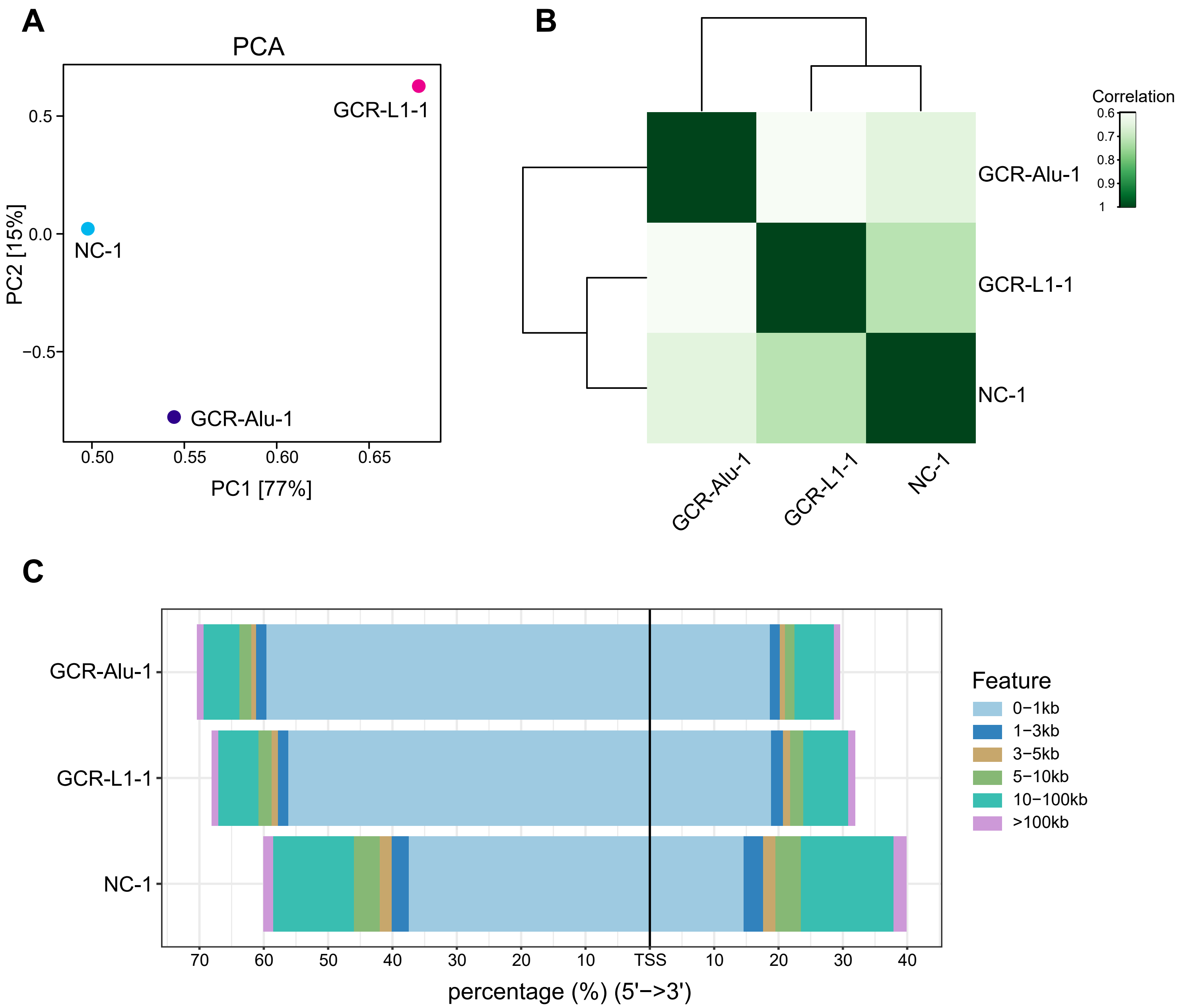

Supplementary Fig. 12

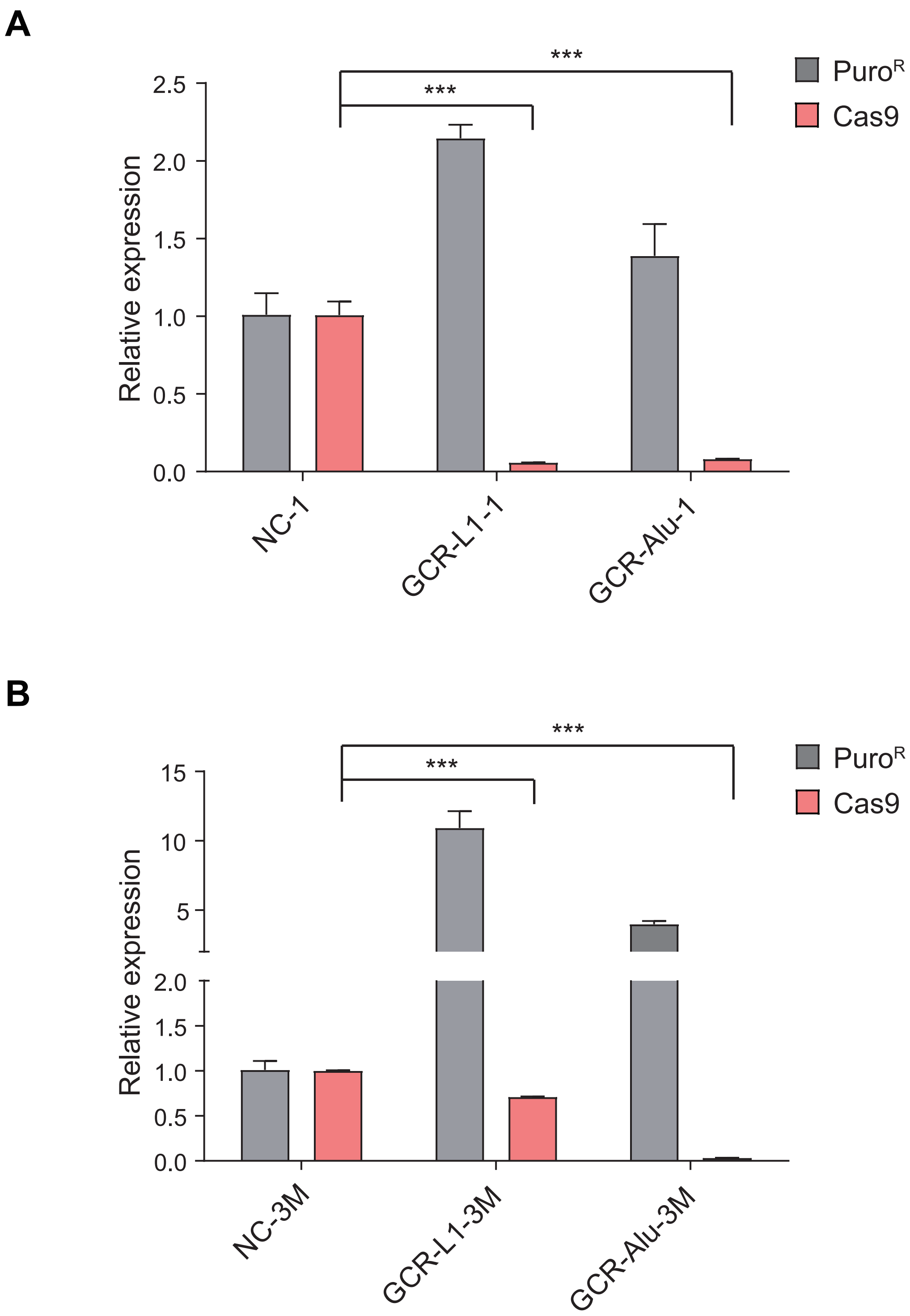

Supplement: gkac153_Supplemental_Files [file gkac153_supplemental_files.zip › Supplementary Materials.pdf]
